# Fluorescent reporter assay reveals ribonucleotides promote mismatch correction *in vivo*

**DOI:** 10.1093/nar/gkag814

**Published:** 2026-08-31

**Authors:** Sophie Erlich, Patrizia Mazzucato, Daniel J Laverty, Leona D Samson, Zachary D Nagel

**Affiliations:** Department of Environmental Health, Harvard T.H. Chan School of Public Health, Boston, MA 02115, United States; Department of Biological Engineering, Massachusetts Institute of Technology, Cambridge, MA 02139, United States; Department of Chemistry, Lehigh University, Bethlehem, PA 18018, United States; Department of Biological Engineering, Massachusetts Institute of Technology, Cambridge, MA 02139, United States; Department of Environmental Health, Harvard T.H. Chan School of Public Health, Boston, MA 02115, United States

## Abstract

Ribonucleotides can serve as a strand discrimination signal in reconstituted *in vitro* biochemical mismatch repair (MMR) assays, but the influence of ribonucleotides on mismatch correction has not been measured directly *in vivo*. We have developed a fluorescence-based host cell reactivation assay that reports correction of a mismatch in proximity of a site-specifically incorporated ribonucleotide. A ribonucleotide leads to enhanced mismatch correction. While neither inactivation of a single allele nor knockdown of RNaseH2 is sufficient to suppress ribonucleotide directed MMR, a modest but statistically significant impairment for repair of mismatches in the presence of an embedded ribonucleotide is observed in RNaseH2 knockout cell lines. Reporter plasmids with ribonucleotides located in either the 3′ or 5′ orientation are robustly repaired in MMR-proficient cells but are weakly repaired in MMR-deficient cells, underscoring their utility as effective MMR reporters. Significant ribonucleotide-enhanced mismatch correction was consistently observed in MMR-deficient cells when the ribonucleotide is in the 3′ orientation. The presence of a ribonucleotide led to enhanced MMR even in RNaseH2 knockout cells, suggesting that other enzymes may promote ribonucleotide-directed MMR. Loss of RNaseH2 was not sufficient to confer significant resistance to the alkylating agent, temozolomide, in support of a model in which ribonucleotide-directed repair events make minor contributions to the canonical MMR pathway in mammalian cells. We propose a model in which MMR-independent ribonucleotide enhanced correction of mismatches can proceed by ribonucleotide excision repair when the ribonucleotide is in the 5′ direction, and proceeds by an unknown mechanism when the ribonucleotide is in the 3′ direction.

## Graphical Abstract

**Graphical Abstract:**
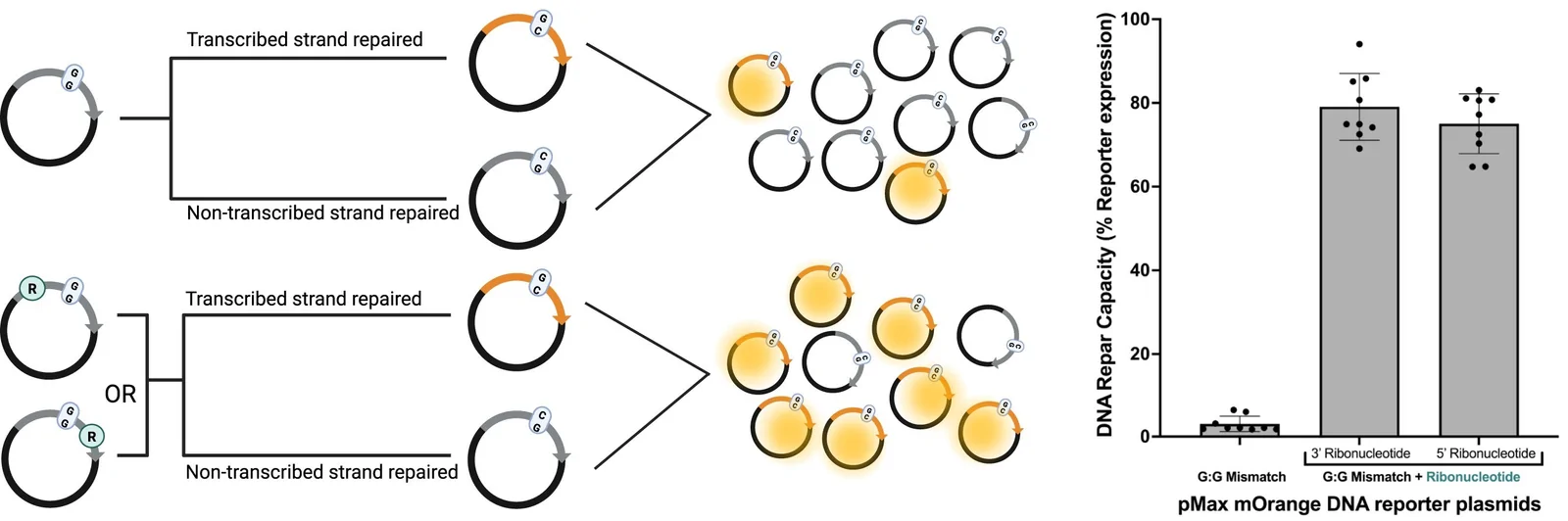

## Introduction

DNA mismatches are a result of errors in DNA replication [1]. It is estimated that DNA polymerases make 10 000 to 100 000 errors each time a mammalian diploid cell replicates its genome [2]. While most errors are corrected by polymerase proofreading activity, mismatches that persist to the second round of DNA replication result in mutations and cancer [3]. The mismatch repair (MMR) pathway is comprised of several multi-protein complexes that recognize and correct mismatches. Over the last three decades, the MMR pathway has been thoroughly characterized and reconstituted *in vitro* [4–7], affording a detailed understanding of the roles of the core MMR proteins (Table 1) [8]. The characterization of these proteins and their function within the MMR pathway in eukaryotes has primarily been conducted using biochemical assays in cell-free extracts and genetic studies of yeast and mouse models [9]. These seminal studies uncovered protein–protein interactions in yeast and MMR activity in human cell extracts to establish the MMR pathway [10, 11].

**Table 1.** Mammalian cell machinery (enzymes, proteins, etc.) involved in the MMR pathway. Each component plays a unique, important role to correct for improper base pairing

MMR components	Responsibility
MutS: MutSα (MSH2-MHS6), MutSβ (MSH2-MHS3)	Recognizes DNA mismatches and loops
MutL: MutLα (MLH1-PMS2), MutLβ (MLH1-PMS1), MutLγ (MLH1-MLH3)	Performs molecular matchmaking, endonuclease activity, and termination of mismatch-provoked excision
RFC	Loads PCNA and aids polymerase function
PCNA	Initiates DNA excision, promoting DNA resynthesis
EXO1	Excises DNA in the newly synthesized strand
polδ	Carries out DNA resynthesis and strand displacement synthesis in the case of EXO1-independent MMR
Ligase I	Ligates nicks following repair synthesis

Despite our thorough understanding of the core MMR machinery, questions remain regarding the mechanisms by which MMR distinguishes between strands for accurate mismatch correction. Early investigations demonstrated that single strand breaks in the newly synthesized strand can serve as the strand discrimination signal, particularly in the lagging strand [12–14]. More recent work has revealed that ribonucleotides, which are incorporated into DNA during replication at a frequency of approximately 1 in 6500 bases, can also serve as a strand discrimination signal [15, 16]. Ribonucleotides are incised by RNaseH2, resulting in a nick in the newly synthesized strand containing the mismatch. Outside the context of MMR, this nick would simply be sealed during the downstream steps of ribonucleotide excision repair (RER), but the single strand break intermediate is also proposed to promote strand-specific MMR [15–18]. Despite clear evidence *in vitro*, this process has not yet been observed directly *in vivo*.

In this study, we evaluate the capacity of cells to correct a single-base mispair with or without an adjacent ribonucleotide using a fluorescent multiplexed host cell reactivation (FM-HCR) assay in human cells [19]. This assay confirms that a ribonucleotide promotes strand-specific repair of mismatches whether they are in the 3′ or 5′ orientation with respect to the ribonucleotide. We further show that ribonucleotide enhanced mismatch correction (REMMC) is only partially dependent on RNaseH2 activity, and significant REMMC can occur in the absence of core MMR factors, when the ribonucleotide is embedded in the 3′ direction from the mismatch. The assay system reported herein provides a new tool for measuring MMR and investigating the molecular mechanisms underlying multiple pathways that contribute to REMMC.

## Materials and methods

### Reagents

Enzymes: All enzymes were purchased from New England Biolabs (NEB).

Antibodies:

MLH1: Cell Signaling Technologies Rabbit clone D38G9, Catalog #4256

MSH2: Cell Signaling Technologies Rabbit D24B5, Catalog #2017

MSH6: BD Transduction Laboratories Mouse Clone 44/MSH6, Catalog #610918

GAPDH: Santa Cruz Biosciences clone 0411, catalog sc-47724

Oligonucleotides:

DNA oligonucleotides for reporter preparations were from Integrated DNA Technologies (IDT). Single-guide RNAs (sgRNAs) for CRISPR–Cas9 knockout were from Thermo Fisher.

### Biological resources

#### Cell lines

Human lymphoblastoid TK6 cells, including WT, RNaseH2A^−/−^, MSH2^−/−^, and MLH1^−/−^, were obtained from the TK6 mutant consortium at the National Institute of Health Sciences in Japan. Primary p53^−/−^ C57BL6/J mouse embryonic fibroblasts were a gift from Martin Reijns characterized previously and confirmed to lack RNaseH2B protein expression [20]. Human tumor cell lines U251, U2OS, HCT116, HCT15, DU145, and SKOV3 were obtained from the National Cancer Institute (NCI-60 panel) Division of Cancer Treatment and Diagnosis. All TK6, HCT116, HCT15, DU145, and SKOV3 cells were maintained with RPMI media supplemented with 10% fetal bovine serum (FBS). U251, U2OS, LN229, and primary p53^−/−^ C57BL6/J mouse embryonic fibroblasts, including WT, heterozygous and homozygous mutants for RNaseH2B were maintained in Dulbecco's modified eagle medium (DMEM) supplemented with 10% FBS. Cell lines were tested for mycoplasma contamination at first recovery and every 6 months throughout experimentation with the Lonza MycoAlert Mycodetection Kit (LT07-218) and found to be negative. Expression of MMR proteins of interest in all cell lines and deficiencies reported previously by others were confirmed by western blot.

#### Generating and validating RNaseH2A knockdown in LN229 cells

Stable knockdown of RNaseH2A was carried out using the green fluorescent protein (GFP) tagged plasmid cytomegalovirus cloning vector (pcDNA) for mammalian expression of small non-coding MicroRNA (miRNA) molecules (full vector name: pcDNA 6.2-GW/EmGFP-miR) in the BLOCK-iT Pol II miR RNAi Expression Vector Kit (Thermo Fisher Scientific). Four optimized candidate miRNAs targeting human RNaseH2A were designed using BLOCK-iT^™^ RNAi Designer software. The candidate miRNAs were cloned into the pcDNA 6.2-GW/EmGFP-miR vector. Transient transfection of LN229 cells with miRNA expressing vectors was carried out using Lipofectamine 2000. A vector expressing a non-targeting control miRNA (NT), one copy of the miRNA that yielded the most efficient knockdown (R1) or two chained copies (R2) was prepared by cloning the following pre-miRNA oligonucleotide duplex into the pcDNA 6.2-GW/EmGFP-miR vector:

Top: TGCTGTTCGCAAACAGCCTTTCCCGCGTTTTGGCC ACTGACTGACGCGGGAAACTGTTTGCGAA

Bottom: CCTGTTCGCAAACAGTTTCCCGCGTCAGTCA GTGGCCAAAACGCGGGAAAGGCTGTTTGCGAAC

This vector was transfected into LN229 cells. Knockdown was verified at 48 h post-transfection using TaqMan RT-qPCR to measure RNaseH2A transcript levels in GFP positive cells isolated by fluorescence activated cell sorting. Clones stably expressing a non-targeting miRNA control or miRNA targeting RNaseH2A were selected using blasticidin, and confirmed using qPCR analysis of RNaseH2A (Supplementary Fig. S1). The RNaseH2A protein level was not measured in LN229 cells because we observed no effect on repair of the 5′ ribonucleotide containing plasmid, whereas we did observe significant effects in both human and murine knockout cell lines.

#### TaqMan RT-qPCR

Approximately 1 × 10^6^ LN229 cells were collected by centrifugation and suspended in 1 ml of TRIZOL reagent. Following extraction with 200 μl of chloroform, the aqueous layer (approximately 500 μl) was transferred to a new tube, combined with one volume of absolute ethanol, and purified using a Qiagen RNAeasy Mini Kit following the manufacturer’s protocol. Complementary DNA (cDNA) was prepared using a Qiagen Omniscript RT kit. An Applied Biosystems Gene Expression Assay with 6-carboxyfluorescein (6-FAM) dye-labeled TaqMan MGB probes targeting human GAPDH (normalization control) or RNaseH2A was used following the protocol recommended by the manufacturer. Residual RNaseH2A expression in the knockdown cell lines was estimated using the ΔΔCT method.

#### Generation of ribonucleotide containing plasmid

**Figure 1.**
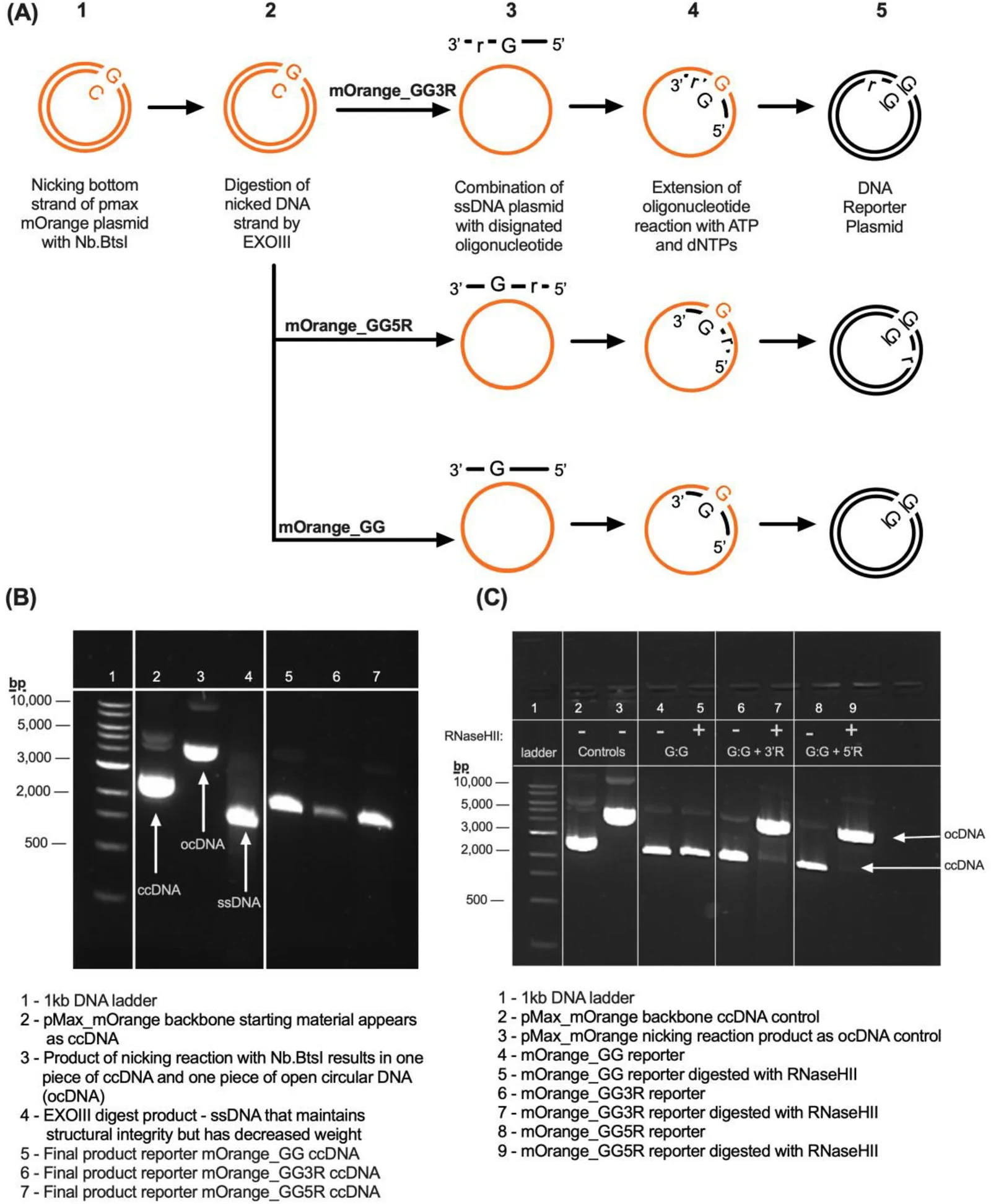
Creating a mismatch correction reporter plasmid that contains a ribonucleotide 50 bp from the lesion on a pMax mOrange backbone. (**A**) The reporter plasmid design and graphical flow of preparation steps leading from the closed circular homoduplex starting material (1), to nicked plasmid (2), ssDNA (3), heteroduplex DNA comprising an oligonucleotide and circular single stranded DNA (4), and the closed circular heteroduplex product (5). The outer, wild-type, coding strand in the diagrams is oriented 5′→3′ in the clockwise direction, and the inner, mutant, transcribed strand runs antiparallel (5′→3′ in the counterclockwise direction). Further plasmid preparation details can be found in (Piett *et al*., 2021). (**B**) Agarose gel electrophoretic analysis of plasmid starting material, intermediates and productrs. (**C**) Agarose gel electrophoretic analysis of product plasmids from analytical digest with recombinant bacterial RNaseHII to confirm ribonucleotide presence.

Reporter plasmids were constructed starting from a pMax_mOrange plasmid that encodes the mOrange fluorescent protein (FP) or a pMax_mPlum plasmid encoding mPlum under a CMV promoter for expression in mammalian cells. Site-specific G:G mismatches were introduced into the mOrange reporter system using a previously described protocol (Fig. 1) [19, 21]. The same strategy was followed for the G:T mismatch containing reporters (not shown in Fig. 1) [22]. This protocol results in the transcribed strand encoding a non-fluorescent mutant protein. If the mismatch is corrected using the non-transcribed strand as a template for repair synthesis, fluorescence is restored. The percentage of plasmids repaired in this manner is estimated from a ratio of ratios that is reported as “% reporter expression.”

Briefly, the transcribed strand of the pMax_mOrange plasmid was nicked using the nicking endonuclease Nb.BstI. Subsequently, exonuclease III digestion yielded single-stranded circular DNA (ssDNA). A synthetic oligonucleotide that introduces a mis-pair at the desired position outlined below were each annealed onto ssDNA followed by an extension reaction with T4 polymerase, ATP, and dNTPs. Finally, T4 ligase sealed the nicked strand, completing resynthesis of the newly generated closed circular double-stranded DNA (ccDNA) plasmid. Confirmation of reporter plasmid preparation steps was assessed using a 1% agarose gel after each step in the preparation process (Fig. 1). Completed product sequence was then confirmed with bidirectional Sanger sequencing (Supplementary Fig. S2)

#### Generation of a plasmid with a 3′ nick 309 nucleotides from a G:G mismatch (mOrange_nGG)

A previously reported procedure was used to generate a nicked heteroduplex (details in Supplementary Fig. S2 of Nagel *et al*., 2014 [21]). Briefly, single-stranded DNA derived from the coding strand of wild-type pMax_mOrange plasmid was generated as described above. The single-stranded DNA was annealed with linearized mutant pMax_mOrange (G299C). This resulted in a heteroduplex with a nick in the transcribed strand located 309 nucleotides from the mismatch in the 3′ direction. The annealed plasmid was treated with plasmid safe ATP-dependent nuclease to eliminate linear and single-stranded DNA and cleaned up by phenol chloroform extraction followed by ethanol precipitation.

#### Oligonucleotide design

Several oligonucleotides were designed to anneal, with a 5′ phosphorylated end, to the region surrounding the 299 position of the pMax_mOrange backbone or the 208 position of the pMax_mPlum backbone. Mismatches were selected based on our previous observation that the G299C mutation in mOrange and the T208C mutation in mPlum result in non-fluorescent proteins [21]. At the 299 position of pMax_mOrange, a G replaced the C on the transcribed strand, opposite a G on the non-transcribed strand creating a G:G mismatch. One oligonucleotide served as a ribonucleotide-free control containing just the G:G mismatch (mOrange_GG); the second included a ribonucleotide 65 bases from the mismatch in the 3′ direction (mOrange_GG3R); and the third included a ribonucleotide 65 bases from the mismatch in the 5′ direction (mOrange_GG5R). At the 209 position of pMax_mPlum, a G replaced A on the transcribed strand, opposite T on the non-transcribed strand, creating a G:T mismatch. One oligonucleotide served as a ribonucleotide-free control containing just the G:T mismatch (mPlum_GT); the second included a ribonucleotide 60 bases from the mismatch in the 5′ direction (mOrange_GT5R); and the third included a ribonucleotide 6 bases from the mismatch in the 5′ direction (mOrange_GT5R*)

|G|= G:G mismatch rC = embedded ribonucleotide

mOrange_GG:

5′ P CTCGAAGTTCATCACG |G| GCTCCCACTTGAAGCC 3′

mOrange_GG3R (3′ ribonucleotide 65 nucleotides from a G:G mismatch):

5′-P CTCGAAGTTCATCACG|G|GCTCCCACTTGAAG CCCTCGGGGAAGGACAGCTTGAAGTAG

TCGGGGATGTCGGCGGGGTGCTT rC ACGTAGGCCTTGGA-3′

mOrange_GG5R (5′ ribonucleotide 65 nucleotides from a G:G mismatch):

5′-P CTTGTAGATGAACT rC GCCGTCCTGCAGGGAGGAGTCCTGGGTCACGGTCACCACGCC

GCCGTCCTCGAAGTTCATCACG |G| GCTCCCACTTGAAGCC-3′

mPlum_GT:

5′-P CACGTAGGCCTTGG|G|GCCGTACATGATCTG-3′

mPlum_GT5R (5′ ribonucleotide 60 nucleotides from a G:T mismatch):

5′-P GCCCT rC GGGGAAGGACAGCTTCAAGTAGTCGGGGATGTC GGCGGGGTGCTTCACGTAGGCCTTGG|G|GCCG TACATGATCTG-3′

mPlum_GT5R* (5′ ribonucleotide 6 nucleotides from a G:T mismatch):

5′-P CACGTAGG rC CTTGG|G|GCCGTACATGATCTG-3′

### FM-HCR assays

DNA repair capacity was measured using the previously described FM-HCR assay [19]. In each experiment, one of the three reporter plasmids described above or an undamaged pMax_mOrange plasmid were transfected into each cell line. Electroporation with the Gene Pulser MXcell^™^ (Bio-Rad) was used to transfect reporter plasmids into TK6 cell lines, and lipid transfection with Lipofectamine^™^ 3000 Reagent (Invitrogen) was used to transfect reporter plasmids into cancer cell lines. All transfections included an undamaged plasmid, pMax_BFP which encodes tagBFP, to control for transfection efficiency. Cells were seeded at 50 000 cells per well with reporter plasmid mixtures transfected into each cell line using their respective transfection method. Following transfection and 24 h of incubation, cells were analyzed by flow cytometry for fluorescent reporter expression. Representative flow cytometry plots illustrating the gating scheme used for the analyses reported herein can be found in Supplementary Fig. S3. The following equations were used to calculate DNA repair capacity:


\begin{eqnarray*}X = \frac{{{\mathrm{Damaged\ mOrange\ Count*\textit{Mean}\ Damaged\ mOrange\ }}{\mathrm{\ Intensity}}}}{{{\mathrm{BFP\ Count*\textit{Mean}\ BFP\ Intensity}}}} \\\end{eqnarray*}

\begin{eqnarray*}Y = \frac{{{\mathrm{Undamaged\ mOrange\ Count*\textit{Mean}\ Undamaged\ mOrange\ Intensity}}}}{{\rm BFP \ {\mathrm{Count*\textit{Mean}\ BFP\ Intensity}}}} \end{eqnarray*}

Percent (%) Reporter expression = *Z* = 100 * $\frac{X}{Y}$

Note: Damaged and undamaged refers to the integrity of the specified reporter plasmid

#### Treating cells with temozolomide

A 25 mM solution of temozolomide (TMZ) was made at the start time of each replicate to ensure freshness of treatment. Serial dilutions were conducted to create 100, 50, 25, 12.5, and 6.25 μM TMZ solutions with RPMI. Five hundred microliters containing 100 000 TK6 WT, MLH1^−/−^, MSH2^−/−^, and RNasH2A^−/−^ were seeded onto 18 wells in 12-well plates, and 500 μl of each TMZ dilution was added to three wells of each cell line to treat cells with designated TMZ concentration or a dimethyl sulfoxide (DMSO) control (0 μM). Cell viability and count was determined using a Vi-CELL Viability Analyzer every 24 h for 72 h to evaluate TMZ toxicity to each cell type over time.

#### Generation of clonal knockout cell lines (MLH1^−/−^ and MSH6^−/−^ clones)

U2OS MLH1^−/−^ and U2OS MSH6^−/−^ cell lines were generated using methods described previously [23]. Briefly, 125 000 U2OS cells were seeded into each well of a 6-well plate and transfected with Cas9 ribonucleoprotein (Cas9 enzyme + sgRNA for desired protein) complexes using Lipofectamine CRISPRMAX (Invitrogen). After 3 days of incubation, cells were collected, counted, and diluted to 10 cells/ml. Hundred microliters of this cell suspension was added to each well of a 96-well plate to seed approximately 1 cell/well. Once single colonies became visible under a microscope (between 7 and 10 days) at 80% confluence, cells were expanded to a 12-well plate, 6-well plate, and finally a T25 flask. Final confirmation of the knockout was determined by collecting ∼1 million cells to extract protein and conduct western blotting protocol as described below:

sgRNA sequences for clonal knockouts

MLH1: ThermoFisher CRISPR1027533_SGM (target DNA sequence: GCGAATATTGTCCACGGTTG)

MSH6: ThermoFisher CRISPR680580_SGM (target DNA sequence: GGAACATTCATCCGCGAGAA

### Western blotting

Protein expression levels of MLH1, MSH2, and MSH6 were validated in all cell lines by western blot. Cells were collected and protein extracts were obtained by lysis with cold NETN buffer (100 mM NaCl, 20 mM Tris, pH 8.0, 0.5 mM ethylenediaminetetraacetic acid, and 0.5% NP-40) containing protease inhibitor cocktail and concentration was determined using the BCA assay kit (Invitrogen). Western blot was conducted as described in our previous work [23]. Briefly, proteins were separated on pre-cast 12% polyacrylamide gels (Bio-Rad) and transferred to a nitrocellulose membrane (Bio-Rad) in cold Tris glycine buffer with 10% methanol at 100 V for 1 h. Membranes were blocked using 7.5% milk in phosphate buffered saline with 0.1% Tween for 1 h and then incubated with 1:1000 dilutions of primary antibody against MLH1, MSH2, or MSH6 in PBST with 5% nonfat milk at 4°C. Twenty-four hours later, blots were washed with PBST and incubated with HRP-conjugated anti-goat or anti-rabbit secondary antibodies (depending on primary antibody origin) (Bio-Rad) diluted in PBST with 5% milk at room temperature for 1 h before washing prior to imaging using Thermo Fisher iBright Get imager. Stripping buffer was used to strip blots (6, 10 min each) followed by washing with PBST. After overnight incubation at 4°C with a 1:2000 dilution (PBST with 5% milk) dilution of primary antibody against GAPDH, blots were washed three times with PBST. Secondary HRP-conjugated secondary (1:2000 dilution) antibody incubation was carried out for 1 h, followed by washing (3×) with PBST, and visualization by chemiluminescence with Clarity Western ECL Substrate (Bio-Rad, cat. no 1705061)

### Antibodies

MLH1: Cell Signaling Technologies Rabbit clone D38G9 (Catalog #4256)

MSH2: Cell Signaling Technologies Rabbit D24B5 (Catalog #2017)

MSH6: BD Transduction Laboratories Mouse Clone 44/MSH6 (Catalog #610918)

### Data collection and statistical analysis

Flow cytometry data were collected with the Attune NxT Flow Cytometer and analyzed using Attune NxT Software Version 2.7. Cell count and fluorescence intensity values were extracted from these data using the calculation outlined above and analyzed using GraphPad Prism version 9.5.0 (525). One-way ANOVA tests with Tukey’s Multiple Comparisons tests were conducted to assess differences in DNA repair capacity of reporter plasmids in the different cell lines.

## Results

### Confirmation assays of reporter plasmid generation

We generated reporter plasmids following our previously reported protocols [19, 21]. The previously described mOrange_GG (GG) reporter plasmid was synthesized in addition to two new reporter plasmids containing a ribonucleotide in the transcribed strand, 65 nucleotides away from the mismatch. One reporter contains a ribonucleotide on the 3′ side of the mismatch (mOrange_GG3R) and the other with a ribonucleotide on the 5′ side of the mismatch (mOrange_GG5R) (Fig. 1A). The purity and closed circular topology of each reporter plasmid preparation step was confirmed by visualizing plasmids following electrophoresis in a 1% agarose gel (Fig. 1B). The presence of a mismatch in the reporter plasmids at the 299th base pair of the reporter gene was confirmed using Sanger Sequencing in the forward and reverse direction of all three reporter plasmids (Supplementary Fig. S2). The presence of a ribonucleotide was confirmed via an analytical digest with recombinant bacterial RNaseHII which resulted in complete conversion of the ribonucleotide-containing mOrange_GG3R and mOrange_GG5R plasmids to an open circular topology but had no effect on the ribonucleotide-free mOrange_GG plasmid (Fig. 1C). Using the same approach, a reporter plasmid with a site specific G:T mismatch at the 208th base pair of the mPlum reporter gene was generated either without a ribonucleotide (mPlum_GT) or with a ribonucleotide on the 5′ side of the mismatch (mPlum_GT5R).

### Ribonucleotides promote correction of single-base mismatches

**Figure 2.**
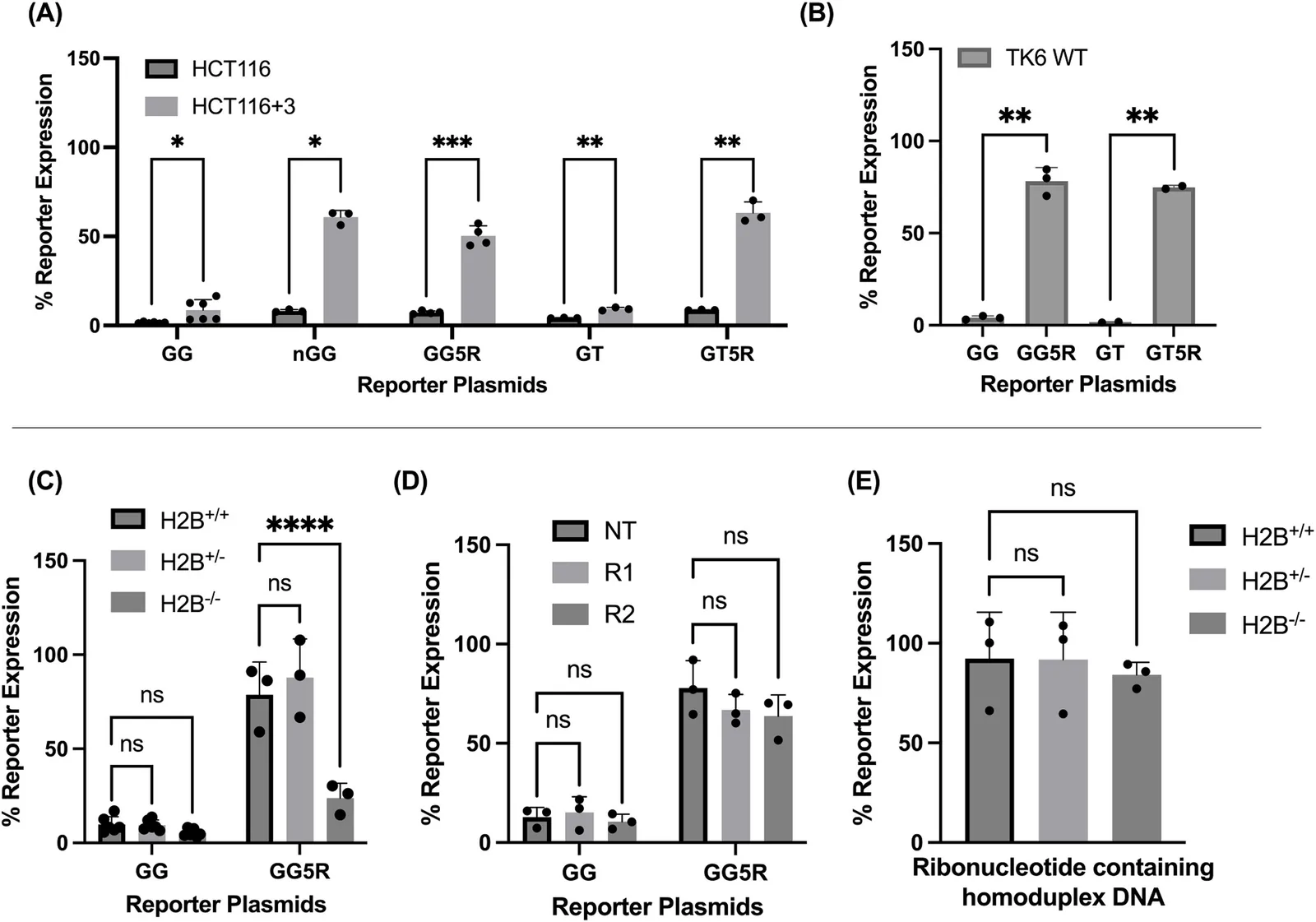
Correction of mismatches in the presence or absence of a 5′ ribonucleotide in the transcribed strand of a fluorescent reporter plasmid. (**A**) The presence of a nick (nGG) or ribonucleotide in the transcribed strand on a G:G mismatch (GG5R) or a G:T mismatch (GT5R) containing reporter plasmid leads to enhanced mismatch correction in human HCT116 cells complemented with chromosome 3 (HCT116 + 3). Statistical significance of differences between cell lines for expression of each reporter plasmid was determined by a paired *t*-test. (**B**) The presence of a ribonucleotide leads to enhanced repair of either a G:G mismatch or a G:T mismatch in TK6 cells, analyzed by paired *t*-tests. (**C**) A ribonucleotide leads to enhanced repair of a G:G mismatch in mouse embryonic fibroblasts in a manner that is partially dependent upon RNaseH2B activity. Analyzed by two-way ANOVA with Dunnett’s multiple comparisons test (**D**) miRNA-mediated knockdown of RNaseH2A in human LN229 cells does not significantly affect repair of a mismatch containing reporter plasmid with an embedded ribonucleotide. NT refers to non-targeting control miRNA; R1 refers to cells expressing a single miRNA against RNaseH2A; R2 refers to cells expressing a chained shRNA with two copies of the targeting sequence. Analyzed by two-way ANOVA with Dunnett’s multiple comparisons test (**E**). Expression in MEFs of ribonucleotide containing homoduplex DNA reported as a percentage of expression for the corresponding fully deoxyribonucleotide homoduplex DNA. Analyzed by one-way ANOVA. Each data point represents an independent biological replicate, and error bars show the standard deviation. In all figure panels, statistical significance is reported as ns (not significant), *P < 0.05, **P< 0.005, ****P< 0.00001

To test whether ribonucleotides promote correction of the G:G mismatch in our reporter plasmids, we transfected mOrange_GG5R into cell lines (Fig. 2).

As expected, based on *in vitro* biochemical assays showing that ribonucleotides can act as a strand discrimination signal in mammalian MMR [15], correction of either a G:G mismatch or a G:T mismatch was dramatically enhanced in MMR-proficient human HCT116 cells complemented with chromosome 3 (HCT116 + 3) when compared to reporter plasmids that lacked a ribonucleotide but were otherwise identical (Fig. 2A). A G:G mismatch reporter with a nick in the transcribed strand (mOrange_nGG), included as a positive control, was repaired with efficiency similar to that of the ribonucleotide containing reporters in HCT116 + 3. For all three reporters, mismatch correction was defective in the MMR-deficient HCT116 cells, confirming that MMR is the dominant pathway reported by the plasmids. Ribonucleotides also strongly enhanced correction of a G:G mismatch and a G:T mismatch in MMR-proficient TK6 cells (Fig. 2B), confirming the phenomenon we refer to as ribonucleotide enhanced mismatch correction (REMMC) *in vivo*.

We next tested whether REMMC depends on the activity of RNaseH2. As expected, RNaseH2B knockout cells, which process ribonucleotides less efficiently, showed significantly diminished mismatch correction (Fig. 2C). Inactivation of a single RNaseH2B allele (H2B^+/−^ in Fig. 2C) did not significantly affect REMMC in MEFS. Expression of a single copy of an miRNA (R1) targeting *RNaseH2A* in human LN229 cells knocked transcript levels down to 12% of the non-targeting control (NT), and expression of two copies (R2) reduced transcript levels to 2% of the control (Supplementary Fig. S1). However, in these cells, as in the H2B^+/−^ MEFS, we did not observe a statistically significant decrease in ribonucleotide-enhanced mismatch correction (Fig. 2D), suggesting that a small amount of RNaseH2 activity is sufficient to support REMMC. In all cases, mismatch correction in the presence of an embedded ribonucleotide or a single strand break exceeded the 50% maximum that is expected and observed for our closed circular mPlum_GT and mOrange_GG reporter plasmids both herein and in our previous publications [24–28]. We confirmed that neither the presence of a ribonucleotide in the transcribed strand of homoduplex DNA nor the status of RNaseH2 significantly affects reporter expression (Fig. 2E). These controls exclude the possibility that the large (2-fold or greater) enhancement of mismatch correction is due to increased expression of ribonucleotide containing plasmids.

**Figure 3.**
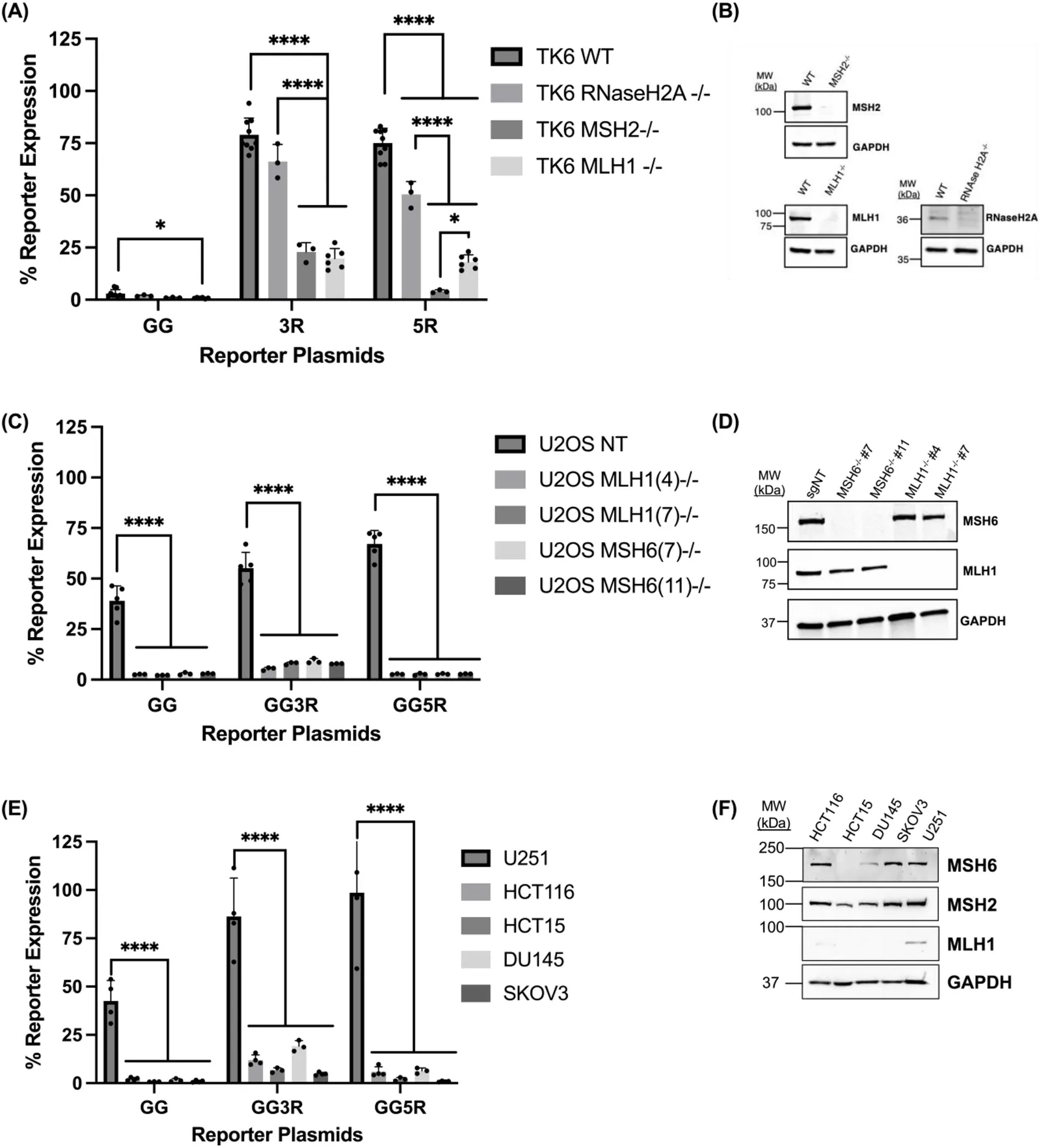
Percent reporter expression for plasmids containing a G:G mismatch (mOrange_GG, GG), and a ribonucleotide 65 nucleotides from the G:G mismatch in the 3′ direction (mOrange_GG3R, 3R) or in the 5′ direction (mOrange_GG5R, 5R). (**A**) Repair of the ribonucleotide containing reporter plasmids is significantly decreased lacking MSH2 or MLH1. Analyzed by one-way ANOVA and Tukey’s multiple comparison’s test. (**B**) Western blot confirming knockout of MSH2, MLH1, and RNaseH2A in TK6 cells. (**C**) Significantly decreased repair of reporter plasmids with mismatches and ribonucleotides in U2OS cells with CRISPR knockouts of MLH1 or MSH6 relative to a non-targeting control (NT). Analyzed by one-way ANOVA and Dunnett’s multiple comparison’s test. (**D**) Western blot confirming knockout of MLH1 or MSH6 in U2OS. (**E**) Deficient repair in four cancer cell lines with previously established MMR defects. Analyzed by one-way ANOVA and Dunnett’s multiple comparison’s test. (**F**) Western blot analysis of MMR protein expression in the cell lines from panel (E). Each data point represents an independent biological replicate, and error bars show the standard deviation. Statistical significance is reported as *P < 0.05, **P < 0.01, ***P < 0.001, ****P < 0.0001 for multiple comparisons test.

To validate and expand upon our initial findings, we generated mOrange_GG5R as well as mOrange_GG3R using our updated protocol that allows the generation of larger amounts of reporter plasmids [19]. Consistent with results in Fig. 2, mismatch correction in the ribonucleotide-containing reporters was dramatically higher in TK6 cells when compared to ribonucleotide lacking mOrange_GG, and a highly significant defect was detected in MMR-deficient TK6 cells lacking expression of MSH2 or MLH1 (Fig. 3A). Depletion of the proteins targeted in the knockout cell lines was confirmed by western blot (Fig. 3B). While this observation further supports that the majority of mismatch correction proceeds through the MMR pathway, the presence of background repair indicates contributions from MMR-independent pathways as well, particularly for the mOrange_GG3R reporter. Also consistent with our results in RNaseH2B^−/−^ MEFs (Fig. 2C), knockout of RNaseH2A in TK6 cells led to partial loss of REMMC for mOrange_GG5R (Fig. 3A). Notably, in WT TK6 cells, there was no significant difference in reporter expression between mOrgange_GG3R and mOrgange_GG5R (*P* = 0.38), suggesting that ribonucleotides can promote MMR regardless of whether they occur on the 3′ side of the mismatch or on the 5′ side of the mismatch. Furthermore, and in contrast to our observations with mOrange_GG both herein and in dozens of other cell lines [21, 25, 26], reporter expression significantly exceeded 50%. If the mismatch in these reporter plasmids is unrepaired, or if the non-transcribed strand is repaired, thereby fixing the non-fluorescent base substitution in the transcribed strand, then a non-fluorescent protein is expressed. Consequently, % reporter expression approximates the percentage of plasmids in which the mismatch has been corrected in the transcribed strand. The data are thus consistent with a model in which ribonucleotides promote MMR in a manner that is strand-specific. Although data have been reported in support of a role for ribonucleotides in MMR, this is, to our knowledge, the first direct demonstration of this role in human cells.

### 3′ Ribonucleotides can promote an MMR independent mismatch correction mechanism

To test verify that REMMC of the G:G mismatch in our reporter plasmids can be attributed to the MMR pathway, we measured repair in seven additional cell lines lacking core MMR proteins, including two independent MSH6 knockouts in U2OS, two MLH1 knockouts in U2OS, and three independently verified MMR-deficient cancer cell lines with mutations in the MMR genes MSH6 (HCT15), MLH1 and PMS2 (DU145), and MLH1 (SKOV3), and we carried out further analyses with MLH1 mutant HCT116 [29–32]. A large and significant defect in mismatch correction was evident in all 10 cell lines lacking essential MMR proteins (Fig. 3) relative to the MMR proficient controls (TK6, U2OS, and U251). However, as noted above for TK6 knockout cell lines and HCT116, we also noted a relatively low level of background mismatch correction for ribonucleotide containing reporter plasmids in some MMR-deficient cell lines. MLH1^−/−^ TK6 cells that are completely unable to repair the ribonucleotide-lacking mOrange_GG reporter partially retained the ability to repair both ribonucleotide-containing reporter plasmids, mOrange_GG5R and mOrange_GG3R, albeit with much lower efficiency (approximately 20%) than MMR proficient TK6 cells (80%). MSH2^−/−^ TK6 cells, which also are unable to repair the mOrange_GG reporter, exhibit weak expression of the mOrange_GG5R reporter (approximately 5%), but repaired the mOrange_GG3R reporter with efficiency similar to the MLH1^−/−^ cells (Fig. 3A and Supplementary Fig. S4). Taken together, the results for mOrange_GG3R suggest that a 3′ ribonucleotide can promote correction of mismatches in a manner that is relatively weak but independent of multiple essential MMR proteins.

In U2OS cell lines with the key MMR genes, MLH1 or MSH6, inactivated by CRISPR–Cas9-mediated knockout (Fig. 3C and D), and in cancer cell lines that lack expression of key MMR proteins (Fig. 3E and F), we consistently observed a strong defect in mismatch correction relative to the repair proficient controls. The efficiency of MMR-independent mismatch correction was cell line dependent, but every MMR defective cell line retained some ability to correct the mismatch in mOrange_GG3R, which exceeded the ability to correct the mismatch in mOrange_GG5R in every case.

### Sensitivity to alkylating agents

**Figure 4.**
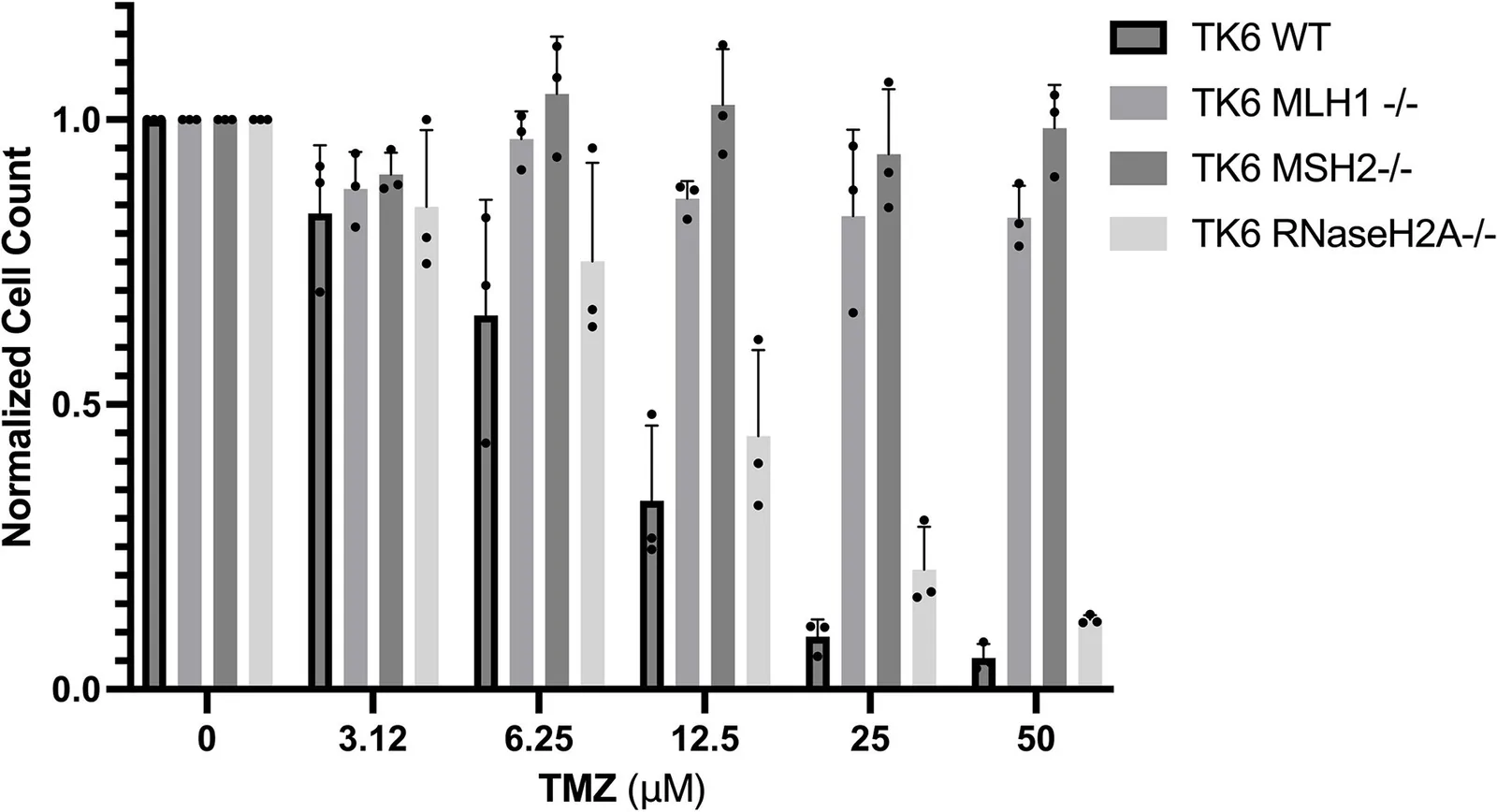
TK6 cells – WT, MLH1^−/−^, MSH2^−/−^, and RNaseH2A^−/−^ were treated with increasing concentrations of TMZ to evaluate alkylating agent resistance in MMR-deficient cells.

Resistance to alkylating agents is a hallmark of MMR deficiency. TK6 cells are particularly sensitive to SN1-like alkylating agents such as TMZ because they do not express methylguanine methyltransferase (MGMT), which repairs cytotoxic *O*^6^-methylguanine lesions induced by TMZ. Loss of MMR in TK6 leads to resistance to SN1-like alkylating agents [33, 34]. As expected, and consistent with these previous findings, MLH1^−/−^, MSH2^−/−^ TK6 cells exhibited marked resistance to TMZ relative to WT (Fig. 4). However, RNaseH2A^−/−^ TK6 cells were not significantly resistant to TMZ when compared to wild-type TK6. We also tested TMZ sensitivity in the RNaseH2B^−/−^ MEFS and found them to be more sensitive to TMZ than WT, likely due to diminished MGMT activity (Supplementary Fig. S5). Since even a minor defect in MMR is sufficient to result in large changes in TMZ sensitivity [27], these data further support a relatively minor contribution of ribonucleotides to the efficiency of MMR in mammalian cells.

## Discussion

**Figure 5.**
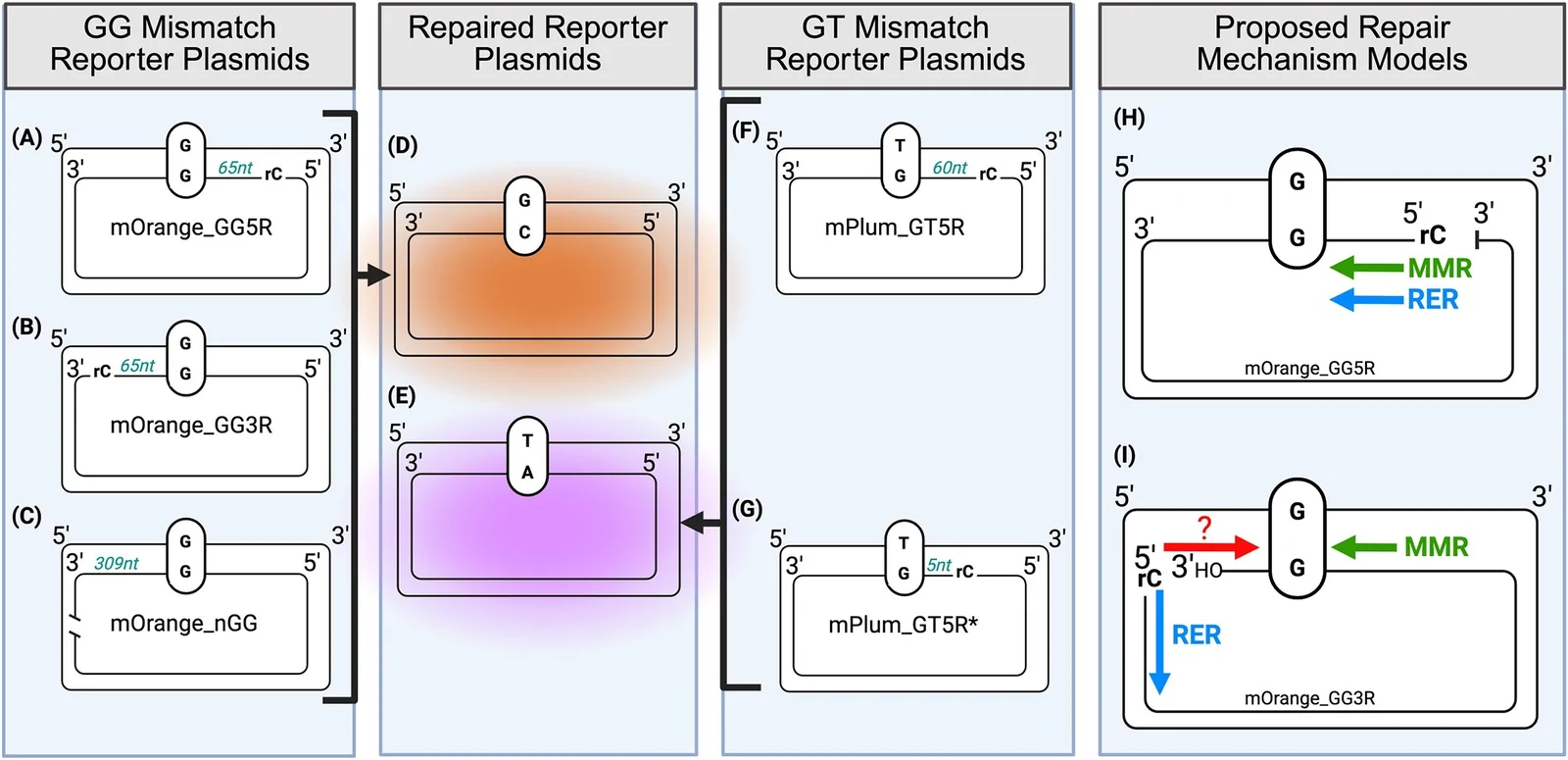
Mismatch reporter plasmid diagrams and models illustrating directionality of key steps in mismatch correction pathways. Plasmids are represented as rounded squares in which the outer solid line represents the coding strand oriented 5′→3′ in the clockwise direction and the inner solid line represents the transcribed template strand oriented 5′→3′ in the counterclockwise direction. (**A**) The mOrange_GG5R reporter plasmid has a ribonucleotide (rC) embedded in the transcribed strand 65 nt (indicated in turquoise) from the GG mismatch (indicated by a bubble marked “G G” crossing both strands) in the 5′ direction. (**B**) The mOrange_GG3R reporter plasmid has a ribonucleotide embedded in the transcribed strand 65 nt from the GG mismatch in the 3′ direction. (**C**) The mOrange_nGG reporter plasmid has a nick in the transcribed strand 309 nt away from the GG mismatch in the 3′ direction. (**D**) The repaired plasmid product that gives rise to an mOrange fluorescent signal is the same for all three GG mismatch reporters (A–C). (**E**) The repaired plasmid product that gives rise to an mPlum fluorescent signal is the same for both GT mismatch reporters (**F** and **G**). (F) The mPlum_GT5R reporter plasmid has a ribonucleotide embedded in the transcribed strand 60 nt from the GT mismatch (indicated by a bubble marked “T G” crossing both strands) in the 5′ direction. (G) The mPlum_GT5R* reporter plasmid has a ribonucleotide embedded in the transcribed strand 5 nt from the GT mismatch in the 5′ direction. (**H**) DNA repair pathways that may correct a mismatch with a 5′ ribonucleotide. Strand removal proceeds in the direction represented by the arrow. Strand displacement DNA synthesis during RER (blue) or strand excision by Exo1 during MMR (MMR, green) can contribute to mismatch correction in the transcribed strand of the reporter plasmid. (**I**) DNA repair pathways that may correct a mismatch with a 3′ ribonucleotide. RER (blue) does not contribute because strand displacement synthesis proceeds away from the mismatch. MMR (MMR, green) requires the action of a nuclease (such as MutL) capable of generating a 5′ strand break, from which Exo1-dependent strand excision can then proceed as in panel (H). Background mismatch correction observed in the absence of MLH1, MSH2, or MSH6 might be promoted by a 3′→5′ exonuclease as part of an unknown alternative mechanism (question mark, red).

We have developed plasmid-based mOrange fluorescent reporter assays with G:G mismatches (Fig. 5A–D) and mPlum fluorescent reporter assays with G:T mismatches (Fig. 5E–G) that robustly detect defects in MMR and provide direct evidence to support a role for ribonucleotides serving as a potent strand discrimination signal for MMR in mammalian cells. Surprisingly, a ribonucleotide can also promote relatively weak but significant correction of a single-base mis-pair in cell lines lacking proteins that are required for MMR, consistent with an alternative mechanism for mismatch correction that does not require all core MMR proteins. The MMR-independent mismatch correction pathway is consistently observed when the ribonucleotide is located in the 3′ orientation relative to the mismatch, excluding a role for RER. In keeping with the widely accepted definition of MMR as a mechanism that strictly requires the core MMR proteins, we have adopted the broader term “mismatch correction” that encompasses both MMR and the repair activity we observe in MMR-deficient cells.

Repair of the newly synthesized daughter DNA strand is essential for accurate MMR, and the introduction of a strand break is a key early step in the repair process. The methyl-directed strand discrimination mechanism that has been firmly established in bacterial model organisms such as *Escherichia coli* does not operate in mammalian cells [15]. Regular strand discontinuities and Okazaki fragments in the lagging strand, proliferating cell nuclear antigen (PCNA)-directed MutLα endonuclease activity, and strand break intermediates of RER [35] are all thought to serve as strand discrimination signals in mammalian cells [36, 37]. Biochemical assays performed using cell extracts demonstrated that a ribonucleotide could promote strand-specific MMR *in vitro* [12, 15]. A second paper published at the same time indicated that ribonucleotides are a strand discrimination signal for MMR in the leading strand [16]. However, ribonucleotide directed MMR has not been directly observed *in vivo*. Our data reveal that ribonucleotides promote strand specific correction of a single-base mispair *in vivo* (Fig. 3).

### Ribonucleotides enhance mismatch correction in the ribonucleotide containing strand independent of the orientation of the ribonucleotides with respect to the mismatch

The closed circular ribonucleotide-lacking mOrange_GG reporter plasmid lacks any known strand discrimination signal and across over 100 cell lines and primary cells we have never observed reporter expression in excess of 50%. This consistent observation suggests the G:G mismatch in mOrange_GG is repaired in a strand-independent manner, since repair of the non-transcribed strand would leave the transcribed strand unchanged and thus would not result in expression of the orange fluorescent reporter protein. Because fluorescent reporter expression requires correction of the mismatch in the transcribed strand, and because we normalize to both a transfection control and a damage-free control, percent reporter expression is expected to closely approximate the fraction of plasmids in which the mismatch has been corrected in the transcribed strand. While closed circular mismatch-containing plasmids are not robustly repaired *in vitro*, they are repaired efficiently in MMR-proficient cells and repair is barely detectable in cells lacking any of the core MMR proteins. When a strand break was included to promote strand-specific repair, repair exceeded 50% as expected, but we also observed a background of mismatch correction in MMR-deficient cells (see nGG, Fig. 2A). Because the primary goal of the original FM-HCR assays was to make accurate and sensitive measurements of MMR *in vivo*, we proceeded with the closed circular reporters that are completely refractory to repair in all cell MMR-deficient cell lines. Here we find that a ribonucleotide results in reporter expression in excess of 50% for all four MMR-proficient mammalian cell lines tested (TK6, U2OS, MEFS, and U251), for both 5′ and 3′ ribonucleotides. Although the assay does not provide direct information regarding the fate of the non-transcribed strand, we conclude that ribonucleotides likely drive mismatch correction in a strand-specific manner *in vivo*. Except in the MLH1^−/−^ TK6 cell line, MMR-independent correction of the mismatch in mOrange_GG5R was either very weak (HCT116, DU145, and MSH2^−/−^ TK6) or altogether absent (MLH1^−/−^ and MSH6^−/−^ U2OS, HCT15, and SKOV3). Based on its ability to distinguish between MMR-proficient and MMR-deficient cells and its efficient (>50%) repair across all cell lines, mOrange_GG5R is a reliable assay for MMR and performs at least as well as mOrange_GG. Since there may be a faint band at the expected molecular weight of MLH1 (Fig. 3B) we cannot rule out the possibility that the partial repair mOrange_GG5R in the MLH1^−/−^ TK6 cell line could be explained by a low level of residual MLH1 protein expression.

### Cells that are deficient for REMMC are not obviously resistant to alkylating agents, implying a minor role in overall MMR

TK6 cells are hypersensitive to alkylating agents because they do not express MGMT. Loss of MSH6 rescues this hypersensitivity [33], and resistance to alkylating agents is considered a hallmark of MMR deficiency. Here we confirm the expected resistance to alkylating agents in the MSH2- and MLH1-deficient TK6 mutants. Our finding that REMMC-deficient RNaseH2A knockout TK6 cells are at most mildly resistant to TMZ indicates that the ribonucleotide-dependent MMR pathway is not likely to affect cancer cell sensitivity to alkylating agents, at least when other strand discrimination signals are available. Our findings in TK6 are consistent with our finding that RNaseH2B knockout MEFS show no evidence of resistance to TMZ; instead TMZ sensitivity was dependent upon MGMT expression, as would be expected in an MMR proficient background. We note, however, that we cannot rule out the possibility that RNaseH2 depletion sensitizes cells to TMZ-induced DNA lesions other than *O*^6^-methylguanine, thereby masking any resistance to the same alkylating agent that would accompany a potential MMR defect. Furthermore, while knockout of either subunit (RNaseH2A or RNaseH2B) is expected to destabilize and inactivate the RNaseH2 obligate heterotrimer, both knockout cell lines retain REMMC activity that could contribute to alkylating agent sensitivity.

### Ribonucleotides can promote mismatch correction in cells that lack MMR proteins

Our data support a strict requirement for MSH2 and MSH6 (all cell lines studied) and a near universal requirement for MLH1 (all cell lines except TK6) for the correction of mismatches with a 5′ ribonucleotide. Strand displacement synthesis during RER [16] initiated by a 5′ ribonucleotide could in principle proceed through the mismatch, leading to MMR-independent mismatch correction (Fig. 5H). Since the repair tract is not expected to be more than a few nucleotides [38], RER-dependent mismatch correction is expected to depend strongly on the distance between the mismatch and the ribonucleotide. Consistent with this model, MMR-independent correction mPlum_GT5R* where the distance was 6 nt was more efficient than for mPlum_GT5R where the distance was 60 nt (Supplementary Fig. S6). Notably, an alternative Exo1-independent mismatch correction pathway involving strand displacement synthesis by Polδ can repair a 5′ mismatch in a manner that is independent of *both* MutLα and MutSα [39, 40]. This Exo1-independent mechanism may also explain the repair of mOrange_GG5R in cell lines lacking key MMR proteins. The mismatch in the ribonucleotide containing reporter plasmid mOrange_GG3R was consistently corrected with greater efficiency than the ribonucleotide-lacking reporter mOrange_GG in MMR-deficient cells. In some instances, when comparing reporter expression of mOrange_GG3R to mOrange_GG, the enhancement of repair was as large as 20-fold (Supplementary Tables S1–S3). While a strand break on either side of the mismatch can direct MMR [41], neither the 5′→3′ hydrolytic activity of ExoI nor the strand displacement activity of a DNA polymerase (likewise 5′→3′) carrying out RER or Exo1-independent mismatch correction can remove the stretch of DNA between a 3′ strand break and the mispair. Consequently, MMR directed by a 3′ break strictly requires the nuclease activity of MutLα, which generates a second break on the distal (5′) side of the mispair that is compatible with the MMR mechanisms outlined above. The repair of mOrange_GG3R in MMR-deficient cell lines, particularly those lacking MutLα, is unexpected and the mechanism merits future study. Collectively, our data suggest the existence of an MMR-independent mismatch correction mechanism that can be provoked by a 3′ ribonucleotide or strand break (Fig. 5I).

### Newly designed reporter plasmids assess the ribonucleotide effect

Two new reporter plasmids, mOrange_GG3R and mOrange_GG5R, were designed and generated for the purposes of this study and provide a platform for future studies aimed at dissecting mechanisms by which a ribonucleotide on the 3′ or 5′ side of a mismatch can influence repair. Like other FM-HCR assays, these reporter systems are highly validated and reliably confirm results that are expected based on gold standard biochemical approaches [19, 27, 42], but hold the advantage of rapid analysis of multiple site-specific DNA lesions in parallel in the physiological context of living cells. A more detailed comparison of the FM-HCR reporter assays with alternative methods has been made in previous publications [19]. In addition to their application to studies focused on understanding the molecular mechanism of MMR, these new reporter systems may provide a powerful strategy for measuring inter-individual differences in MMR in human populations [43]. Since responses to both alkylating agents and immune checkpoint blockade are dependent upon the status of MMR [44, 45], these assays could also potentially be applied in tumor biopsies to reveal the MMR status of primary cancers and support personalized treatment decisions.

### Study limitations and strengths

While our FM-HCR reporters can directly evaluate the effect of a ribonucleotide on MMR *in vivo*, the assay is subject to some important limitations. First, although plasmids are incorporated onto nucleosomes and FM-HCR assays are in some cases sensitive to the biological effects of disrupting chromatin remodeling proteins [46], they are not configured to represent the full complexity of chromatin. In addition, being based on the expression of a fluorescent reporter gene, the FM-HCR assay is inherently limited to providing information about the DNA strand that is transcribed. Consequently, the assays cannot assess the effects of ribonucleotides on MMR in the non-transcribed strand or MMR in inactive genes. Additionally, this study only evaluated MMR in a few immortalized cell lines, which could limit the generalizability of this knowledge to all tissue types and primary cells. We only investigated cells lacking single factors, MMR factors or RNaseH2, restricting our ability to evaluate potential protein deficient interactions and generalizability to all cell tissue further. Beyond the novelty of this protocol, strengths of this study include the ability to study REMMC *in vivo*, the ability to control the exact location of the mismatch and the ribonucleotide, and the use of multiple cell lines to confirm our observations.

### Future directions

These functional assays provide a starting point for exploring biological questions using chemically defined MMR substrates with physiologically relevant strand discrimination signals *in vivo*. Future studies in human genetic models of MMR deficiency are warranted to explore the extent to which the 3′ ribonucleotide might provoke ExoI-independent and DNA2-dependent MMR or another as yet unknown MMR-independent mismatch correction mechanism. In addition, it would be of interest to determine whether ribonucleotides are similarly potent strand discrimination signals for the repair of insertion/deletion loops. Incorporating these reporters in ongoing population studies and cancer research investigating the links between DNA repair capacity and drug sensitivity represent additional frontiers for future exploration. More broadly, assays that assess genome instability in chromatin in REMMC-deficient cells will be important for validating the physiological relevance of functional relationships that emerge from FM-HCR assays.

## Supplementary Material

gkag814_Supplemental_File

## Data Availability

All data are available within the manuscript and in the Supplementary File.
