# Supplementary material for: Fluorescent reporter assay reveals ribonucleotides promote mismatch correction in vivo
Source: Nucleic Acids Res. 2026 Aug 31;54(16):gkag814. doi: 10.1093/nar/gkag814 (PMC13527164; doi:10.1093/nar/gkag814)
Supplement: Supplementary file 1 [file gkag814_supplemental_file.pdf]

Fluorescent reporter assay reveals ribonucleotides promote mismatch correction *in vivo*

Authors:
Sophie Erlich[a], Patrizia Mazzucato[b], Daniel J. Laverty[a], Leona D. Samson[b], Zachary D. Nagel[a]*

[a] Department of Environmental Health, Harvard T.H. Chan School of Public Heallth, Boston, MA, 02115, USA.
[b] Department of Biological Engineering, Massachusetts Institute of Technology, Cambridge, MA, 02139, USA
[*] Corresponding Author

Corresponding author email: znagel@hsph.harvard.edu

Supplemental Figures and Tables

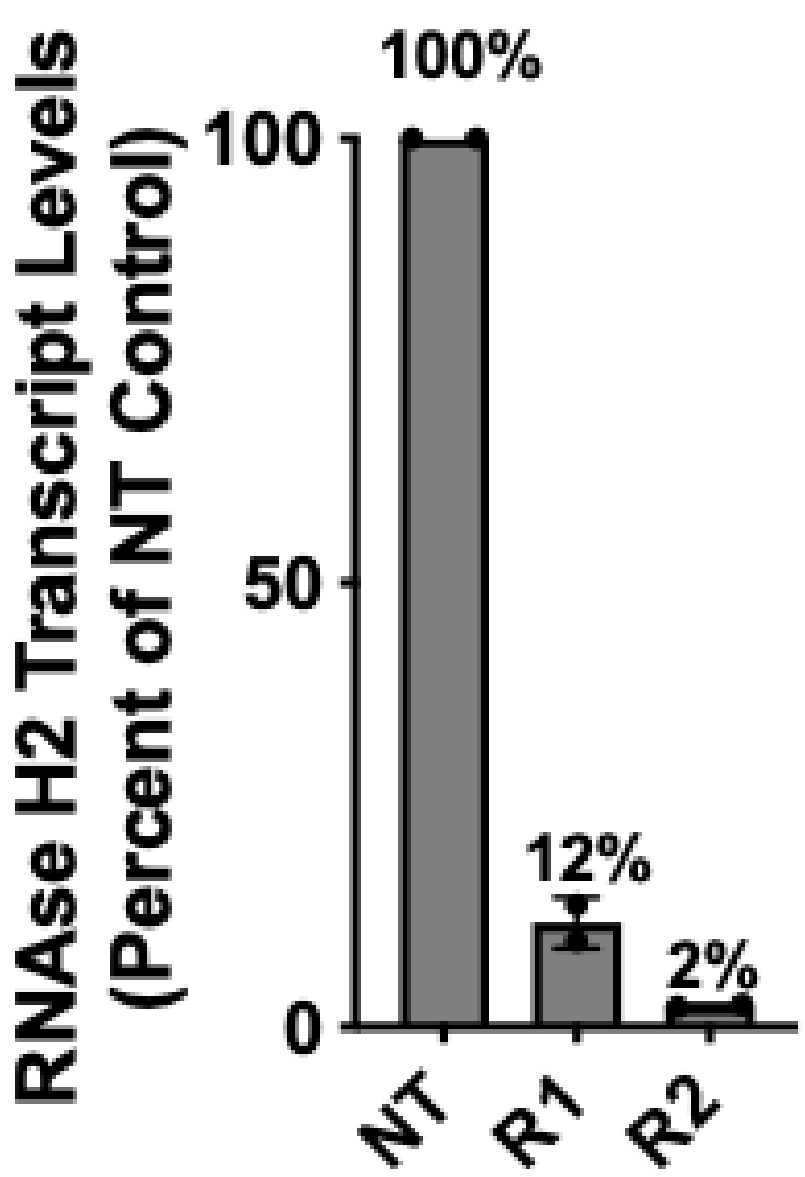

Figure S.1 – Verification of miRNA-mediated knockdown of RNASEH2 in LN229 cells. Transcript levels measured by RT-qPCR are normalized to the non-targeting control (NT), which corresponds to 100%. R1 refers to cells expressing a single copy of the miRNA targeting RNASEH2 and R2 refers to cells expressing two copies of the same miRNA. Normalized gene expression is reported for R1 and R2 for an average of two independent measurements.

Reverse complement sequences of reporter plasmids:

A mOrange_GG

```
>F_GG_pmax_mOrange-CMV-Forward_A05.ab1
NNTTGTACAGNTCGTCCATGCCGCCGGTGGAGTGGCGGCCCTCGGCGCGTTCGTACTGTTCCACGATGGTGTAGTCCTCGTTGTGG
GAGGTGATGTCCAACTTGATGCCGACGATGTAGGCGCCGGGCAGCTGCACGGGCTTCTTGGCCTTGTAGGTGGTCTTGACCTCGGA
GGTGTAGTGGCCGCCGTCCTTCAGCTTCAGCCTCATCTTGATCTCGCCCTTCAGGGCGCCGTCCTCGGGGTACATCCGCTCGGAGG
AGGCCTCCCAGCCCATGGTCTTCTTCTGCATTACGGGGCCGTCGGAGGGGAAGTTGGTGCCGCGCAGCTTCACCTTGTAGATGAAC
TCGCCGTCCTGCAGGGAGGAGTCCTGGGTCACGGTCACCACGCCGCCGTCCTCGAAGTTCATCACGGGCTCCCACTTGAAGCCCTC
GGGGAAGGACAGCTTGAAGTAGTCGGGGATGTCGGCGGGGTGCTTCACGTAGGCCTTGGAGCCGTAGGTGAACTGAGGGGACAGGA
TGTCCCAGGCGAAGGGCAGGGGGCCACCCTTGGTCACCTTCAGCTTAGCGGTCTGAAAGCCCTCGTAGGGGCGGCCCTCGCCCTCG
CCCTCGATCTCGAACTCGTGGCCGTTCACGGAGCCCTCCATGCGCACCTTGAAGCGCATGAACTCCTTGATGATGGCCATGTTATT
CTCCTCGCCCTTGCTCACCATGGTGGCAGCGCTAGCGGTACCCCTGTGGAGAGAAAGGCAAAGTGGATGTCAGTAAGACCAATAGG
TGCCTATCAGAAACGCAAGAATCTTCTCTGTCTCGACAAGCCCAGTTTCTATTGGCCTCCTTAAACCTGTCTTGTAACCTTGATAC
TTACCTGTGATCAGTCGAGCACTGACTGCGTTAGCAATTTAACTGTGATAAACTACCGCAATAAAGCTACTAGTGATCTGACGGTT
CACTAAACGACCTCTGNNNNANNNNNNNNNNNNN
```

mOrange_GG3R

```
>F_3R_GG_pmax_mOrange-CMV-Forward_B05.ab1
NAACTCATCGAGCTCAAGCTTNTACTNGTACAGCTCGTCCATGCCGCCGGTGGAGTGGCGGCCCTCGGCGCGTTCGTACTGTTCCA
CGATGGTGTAGTCCTCGTTGTGGGAGGTGATGTCCAACTTGATGCCGACGATGTAGGCGCCGGGCAGCTGCACGGGCTTCTTGNCC
TTGTAGGTGGTCTTGACCTCGGAGGTGTAGTGGCCGCCGTCCTTCAGCTTCAGCCTCATCTTGATCTCGCCCTTCAGGGCGCCGTC
CTCGGGGTACATCCGCTCGGAGGAGGCCTCCCAGCCCATGGTCTTCTTCTGCATTACGGGGCCGTCGGAGGGGAAGTTGGTGCCGC
GCAGCTTCACCTTGTAGATGAACTCGCCGTCCTGCAGGGAGGAGTCCTGGGTCACGGTCACCACGCCGCCGTCCTCGAAGTTCATC
ACGGGCTCCCACTTGAAGCCCTCGGGGAAGGACAGCTTGAAGTAGTCGGGGATGTCGGCGGGGTGCTNTACGTAGGCCTTGGAGCC
GTAGGTGAACTGAGGGGACAGGATGTCCCAGGCGAAGGGCAGGGGGCCACCCTTGGTCACCTTCAGCTTAGCGGTCTGAAAGCCCT
CGTAGGGGCGGCCCTCGCCCTCGCCCTCGATCTCGAACTCGTGGCCGTTCACGGAGCCCTCCATGCGCACCTTGAAGCGCATGAAC
TCCTTGATGATGGCCATGTTATTCTCCTCGCCCTTGCTCACCATGGTGGCAGCGCTAGCGGTACCCCTGTGGAGAGAAAGGCAAAG
TGGATGTCAGTAAGACCAATAGGTGCCTATCAGAAACGCAAGAATCTTCTCTGTCTCGACAAGCCCAGTTTCTATTGGCCTCCTTA
AACCTGTCTTGTAACCTTGATACTTACCTGTGATCAGTCGAGCACTGACTGCGTTAGCAATTTAACTGTGATAAACTACCGCAATA
AAGCTACTAGTGATCTGACGNTCACTAAACGACCTCTGCNNNNNNNNNNNNNNNN
```

mOrange_GG5R

```
>F_5R_GG_pmax_mOrange-CMV-Forward_C05.ab1
NGTGGTTNGTCCAAACTCATNNNNNNTCAAGCTTNTACTTGTACAGNTCGNCCATGCCGCCGGTGGAGTGGCGGCCCTCGGCGCGTT
CGTACTGTTCCACGATGGTGTAGTCCTCGTTGTGGGAGGTGATGTCCAACTTGATGCCGACGATGTAGGCGCCGGGCAGCTGCACG
GGCTTCTTGGCNTGTAGGTGGTCTTGACCTCGGAGGTGTAGTGGCCGCCGTCCTTCAGCTTCAGCCTCATCTTGATCTCGCCCTTC
AGGGCGCCGTCCTCGGGGTACATCCGCTCGGAGGAGGCCTCCCAGCCCATGGTCTTCTTCTGCATTACGGGGCCGTCGGAGGGGAA
GTTGGTGCCGCGCAGCTTCACCTTGTAGATGAACTCGCCGTCCTGCAGGGAGGAGTCCTGGGTCACGGTCACCACGCCGCCGTCCT
CGAAGTTCATCACGGGCTCCCACTTGAAGCCCTCGGGGAAGGACAGCTTGAAGTAGTCGGGGATGTCGGCGGGGTGCTTCACGTAG
GCCTTGGAGCCGTAGGTGAACTGAGGGGACAGGATGTCCCAGGCGAAGGGCAGGGGGCCACCCTTGGTCACCTTCAGCTTAGCGGT
CTGAAAGCCCTCGTAGGGGCGGCCCTCGCCCTCGCCCTCGATCTCGAACTCGTGGCCGTTCACGGAGCCCTCCATGCGCACCTTGA
AGCGCATGAACTCCTTGATGATGGCCATGTTATTCTCCTCGCCCTTGCTCACCATGGTGGCAGCGCTAGCGGTACCCCTGTGGAGA
GAAAGGCAAAGTGGATGTCAGTAAGACCAATAGGTGCCTATCAGAAACGCAAGAATCTTCTCTGTCTCGACAAGCCCAGTTTCTAT
TGGCCTCCTTAAACCTGTCTTGTAACCTTGATACTTACCTGTGATCAGTCGAGCACTGACTGCGTTAGCAATTTAACTGTGATAAA
CTACCGCAATAAAGCTACTAGTGATCTGACGGTTCACTAAACGACCTCTGNNNNNNNNNNCNNNNNN
```

mOrange_GG3R (additional batch)

```
>F_3R2_GG_pmax_mOrange-CMV-Forward_D05.ab1
NTTNTCCAAANTCATCGAGCTCAAGCTTNTACTNGTACAGNTNGNCCATGCCGCCGGTGGAGTGGCGGCCCTCGGCGCGTTCGTAC
TNTCCACGATGGTGTAGTCCTCGTTGTGGGAGGTGATGTCCAACTTGATGCCGACGATGTAGGCGCCGGGCAGCTGCACGGGCTTC
TTGGCCTTGTAGGTGGTCTTGACCTCGGAGGTGTAGTGGCCGCCGTCCTTCAGCTTCAGCCTCATCTTGATCTCGCCCTTCAGGGC
GCCGTCCTCGGGGTACATCCGCTCGGAGGAGGCCTCCCAGCCCATGGTCTTCTTCTGCATTACGGGGCCGTCGGAGGGGAAGTTGG
TGCCGCGCAGCTTCACCTTGTAGATGAACTCGCCGTCCTGCAGGGAGGAGTCCTGGGTCACGGTCACCACGCCGCCGTCCTCGAAG
TTCATCACGGGCTCCCACTTGAAGCCCTCGGGGAAGGACAGCTTGAAGTAGTCGGGGATGTCGGCGGGGTGCTTCACGTAGGCCTT
GGAGCCGTAGGTGAACTGAGGGGACAGGATGTCCCAGGCGAAGGGCAGGGGGCCACCCTTGGTCACCTTCAGCTTAGCGGTCTGAA
AGCCCTCGTAGGGGCGGCCCTCGCCCTCGCCCTCGATCTCGAACTCGTGGCCGTTCACGGAGCCCTCCATGCGCACCTTGAAGCGC
ATGAACTCCTTGATGATGGCCATGTTATTCTCCTCGCCCTTGCTCACCATGGTGGCAGCGCTAGCGGTACCCCTGTGGAGAGAAAG
GCAAAGTGGATGTCAGTAAGACCAATAGGTGCCTATCAGAAACGCAAGAATCTTCTCTGTCTCGACAAGCCCAGTTTCTATTGGCC
TCCTTAAACCTGTCTTGTAACCTTGATACTTACCTGTGATCAGTCGAGCACTGACTGCGTTAGCAATTTAACTGTGATAAACTACC
GCAATAAAGCTACTAGTGATCTGACGGTTCACTAAACGACCTCTGNNNNNNNNNNCNNNNNN
```

B mOrange_GG

```
>R_GG_pmax_mOrange-CMV-Reverse_A10.ab1
NNGTAAGTATCAAGGTTACAAGACAGGTTTAAGGAGNCCAATAGAAACTGGGCTTGTCGAGACAGAGAAGATTCTTGCGTTTCTGA
TAGGCACCTATTGNTCTTACTGACATCCACTTTGCCTTTCTCTCCACAGGGGTACCGCTAGCGCTGCCACCATGGTGAGCAAGGGC
GAGGAGAATAACATGGCCATCATCAAGGAGTTCATGCGCTTCAAGGTGCGCATGGAGGGCTCCGTGAACGGCCACGAGTTCGAGAT
CGAGGGCGAGGGCGAGGGCCGCCCCTACGAGGGCTTTCAGACCGCTAAGCTGAAGGTGACCAAGGGTGGCCCCCTGCCCTTCGCCT
GGGACATCCTGTCCCCTCAGTTCACCTACGGCTCCAAGGCCTACGTGAAGCACCCCGCCGACATCCCCGACTACTTCAAGCTGTCC
TTCCCCGAGGGCTTCAAGTGGGAGCGCGTGATGAACTTCGAGGACGGCGGCGTGGTGACCGTGACCCAGGACTCCTCCCTGCAGGA
CGGCGAGTTCATCTACAAGGTGAAGCTGCGCGGCACCAACTTCCCCTCCGACGGCCCCGTAATGCAGAAGAAGACCATGGGCTGGG
AGGCCTCCTCCGAGCGGATGTACCCCGAGGACGGCGCCCTGAAGGGCGAGATCAAGATGAGGCTGAAGCTGAAGGACGGCGGCCAC
TACACCTCCGAGGTCAAGACCACCTACAAGGCCAAGAAGCCCGTGCAGCTGCCCGGCGCCTACATCGTCGGCATCAAGTTGGACAT
CACCTCCCACAACGAGGACTACACCATCGTGGAACAGTACGAACGCGCCGAGGGCCGCCACTCCACCGGCGGCATGGACGAGCTGT
ACAAGTAGAAGCTTGAGCTCGATGAGTTTGGACAAACCNCAACTAGAANNNNNNNNNNNNNNNNNNNN
```

mOrange_GG3R

```
>R_3R_pmax_mOrange-CMV-Reverse_B10.ab1
NNNNNNAAGCNNNGTCGTTTAGTGAACCGTCAGATCNNTAGTAGCTTTATTGCGGTAGTTNATCACAGTNAAATTGCTAACGCAGT
CAGTGCTCGACTGATCACAGGTAAGTATCAAGGTTACAAGACAGGTTTAAGGAGNCCAATAGAAACTGGGCTTGTCGAGACAGAGA
AGATTCTTGCGTTTCTGATAGGCACCTATTGNTCTTACTGACATCCACTTTGCCTTTCTCTCCACAGGGGTACCGCTAGCGCTGCC
ACCATGGTGAGCAAGGGCGAGGAGAATAACATGGCCATCATCAAGGAGTTCATGCGCTTCAAGGTGCGCATGGAGGGCTCCGTGAA
CGGCCACGAGTTCGAGATCGAGGGCGAGGGCGAGGGCCGCCCCTACGAGGGCTTTCAGACCGCTAAGCTGAAGGTGACCAAGGGTG
GCCCCCTGCCCTTCGCCTGGGACATCCTGTCCCCTCAGTTCACCTACGGCTCCAAGGCCTACGTGAAGCACCCCGCCGACATCCCC
GACTACTTCAAGCTGTCCTTCCCCGAGGGCTTCAAGTGGGAGCGCGTGATGAACTTCGAGGACGGCGGCGTGGTGACCGTGACCCA
GGACTCCTCCCTGCAGGACGGCGAGTTCATCTACAAGGTGAAGCTGCGCGGCACCAACTTCCCCTCCGACGGCCCCGTAATGCAGA
AGAAGACCATGGGCTGGGAGGCCTCCTCCGAGCGGATGTACCCCGAGGACGGCGCCCTGAAGGGCGAGATCAAGATGAGGCTGAAG
CTGAAGGACGGCGGCCACTACACCTCCGAGGTCAAGACCACCTACAAGGCCAAGAAGCCCGTGCAGCTGCCCGGCGCCTACATCGT
CGGCATCAAGTTGGACATCACCTCCCACAACGAGGACTACACCATCGTGGAACAGTACGAACGCGCCGAGGGCCGCCACTCCACCG
GCGGCATGGACGAGCTGTACAAGTAGAAGCTTGAGCTCGATGAGTTTGGACAAACCACAACTAGAATGCNNGANNNNNNNNNNNNN
NNNNNNNNC
```

mOrange_GG5R

```
>R_5R_pmax_mOrange-CMV-Reverse_C10.ab1
CGCAGTCAGTGCTCGACTGATCACAGGTAAGTATCAAGGTTACAAGACAGGTTTAAGGAGNCCAATAGAAACTGGGCTTGTCGAGA
CAGAGAAGATTCTTGCGTTTCTGATAGGCACCTATTGGTCTTACTGACATCCACTTTGCCTTTCTCTCCACAGGGGTACCGCTAGC
GCTGCCACCATGGTGAGCAAGGGCGAGGAGAATAACATGGCCATCATCAAGGAGTTCATGCGCTTCAAGGTGCGCATGGAGGGCTC
CGTGAACGGCCACGAGTTCGAGATCGAGGGCGAGGGCGAGGGCCGCCCCTACGAGGGCTTTCAGACCGCTAAGCTGAAGGTGACCA
AGGGTGGCCCCCTGCCCTTCGCCTGGGACATCCTGTCCCCTCAGTTCACCTACGGCTCCAAGGCCTACGTGAAGCACCCCGCCGAC
ATCCCCGACTACTTCAAGCTGTCCTTCCCCGAGGGCTTCAAGTGGGAGCGCGTGATGAACTTCGAGGACGGCGGCGTGGTGACCGT
GACCCAGGACTCCTCCCTGCAGGACGGCGAGTTCATCTACAAGGTGAAGCTGCGCGGCACCAACTTCCCCTCCGACGGCCCCGTAA
TGCAGAAGAAGACCATGGGCTGGGAGGCCTCCTCCGAGCGGATGTACCCCGAGGACGGCGCCCTGAAGGGCGAGATCAAGATGAGG
CTGAAGCTGAAGGACGGCGGCCACTACACCTCCGAGGTCAAGACCACCTACAAGGCCAAGAAGCCCGTGCAGCTGCCCGGCGCCTA
CATCGTCGGCATCAAGTTGGACATCACCTCCCACAACGAGGACTACACCATCGTGGAACAGTACGAACGCGCCGAGGGCCGCCACT
CCACCGGCGGCATGGACGAGCTGTACAAGTAGAAGCTTGAGCTCGATGAGTTTGGACAAACCACAACTAGAATGCNNNNNNNNNNN
NNNNN
```

mOrange_GG3R (additional batch)

```
>R_3R2_pmax_mOrange-CMV-Reverse_D10.ab1
NCGTTTAGNNGAACCCGTCAGATCACTAGTAGCTTTATTGCGGTANTTNATCNCAGTNAANTGCTAACGCAGTCAGTGCTCGACTG
ATCACAGGTAAGTATCAAGGTTACAAGACAGGTTTAAGGAGNCCAATAGAAACTGGGCTTGTCGAGACAGAGAAGATTCTTGCGTT
TCTGATAGGCACCTATTGGTCTTACTGACATCCACTTTGCCTTTCTCTCCACAGGGGTACCGCTAGCGCTGCCACCATGGTGAGCA
AGGGCGAGGAGAATAACATGGCCATCATCAAGGAGTTCATGCGCTTCAAGGTGCGCATGGAGGGCTCCGTGAACGGCCACGAGTTC
GAGATCGAGGGCGAGGGCGAGGGCCGCCCCTACGAGGGCTTTCAGACCGCTAAGCTGAAGGTGACCAAGGGTGGCCCCCTGCCCTT
CGCCTGGGACATCCTGTCCCCTCAGTTCACCTACGGCTCCAAGGCCTACGTGAAGCACCCCGCCGACATCCCCGACTACTTCAAGC
TGTCCTTCCCCGAGGGCTTCAAGTGGGAGCGCGTGATGAACTTCGAGGACGGCGGCGTGGTGACCGTGACCCAGGACTCCTCCCTG
CAGGACGGCGAGTTCATCTACAAGGTGAAGCTGCGCGGCACCAACTTCCCCTCCGACGGCCCCGTAATGCAGAAGAAGACCATGGG
CTGGGAGGCCTCCTCCGAGCGGATGTACCCCGAGGACGGCGCCCTGAAGGGCGAGATCAAGATGAGGCTGAAGCTGAAGGACGGCG
GCCACTACACCTCCGAGGTCAAGACCACCTACAAGGCCAAGAAGCCCGTGCAGCTGCCCGGCGCCTACATCGTCGGCATCAAGTTG
GACATCACCTCCCACAACGAGGACTACACCATCGTGGAACAGTACGAACGCGCCGAGGGCCGCCACTCCACCGGCGGCATGGACGA
GCTGTACAAGTAGAAGCTTGAGCTCGATGAGTTTGGACAAACCACAACTAGAATGCNNNNNNNANNNNNNNN
```

Figure S.2: Sanger sequencing confirmation of reporter plasmid sequences. Reverse complement of (A) sequences read in the forward direction and (B) sequences read in the reverse direction to confirm presence of G:G mismatch (highlighted)

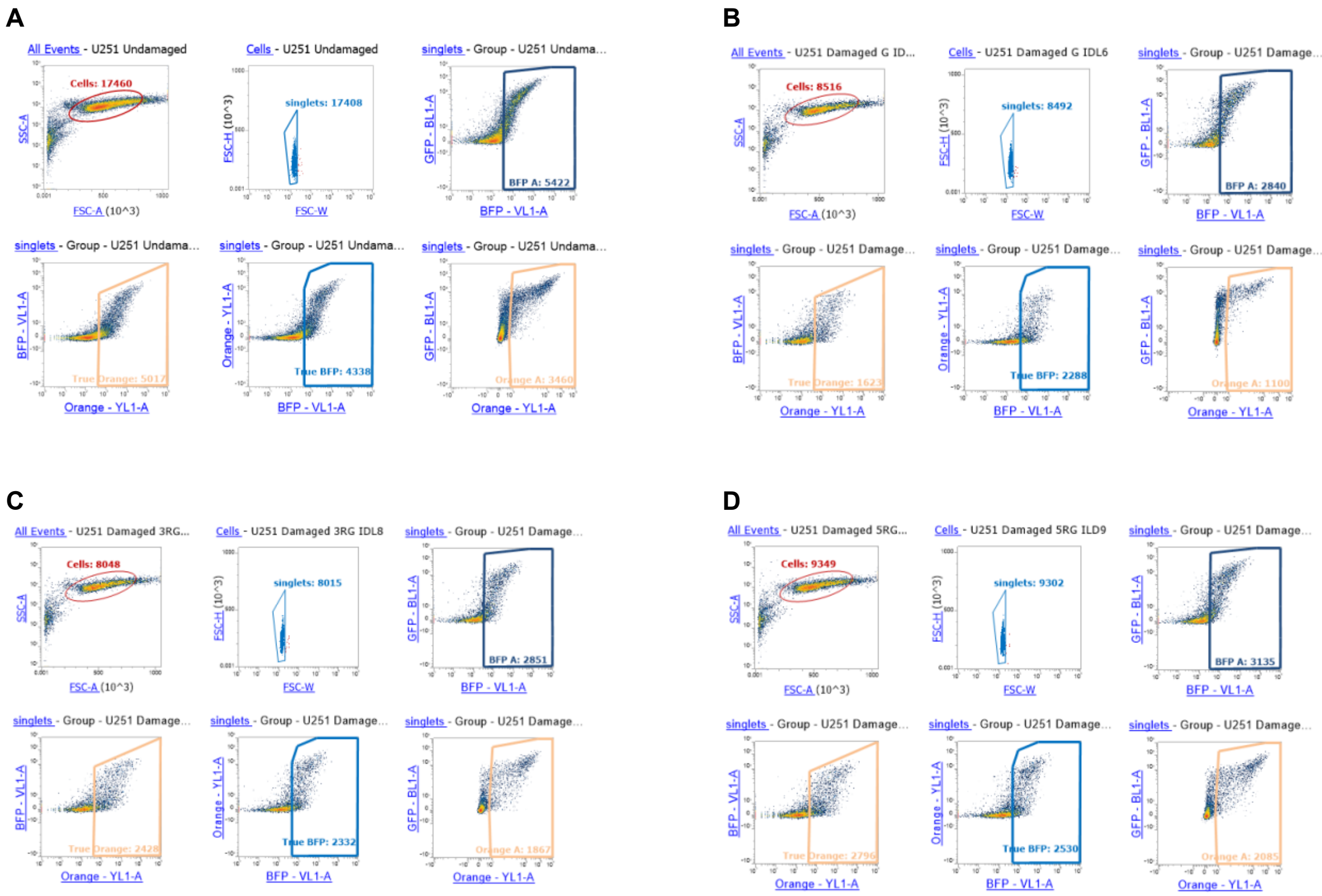

Figure S.3 – Flow cytometry of reporter plasmids transfected into WT U251 Cells. This is an example of a single experiment transfecting the mOrange_GG, mOrange_GG3R, and mOrange_GG5R in WT U251 cells. Additional experiments conducted at the same time using a GFP reporter are also included here. **(A)** shows the transfection data of the undamaged control reporter plasmids, **(B)** shows the data from the mOrange_GG reporter, **(C)** shows the data from the mOrange_GG3R reporter, and **(D)** shows the data from the mOrange_GG5R reporter.

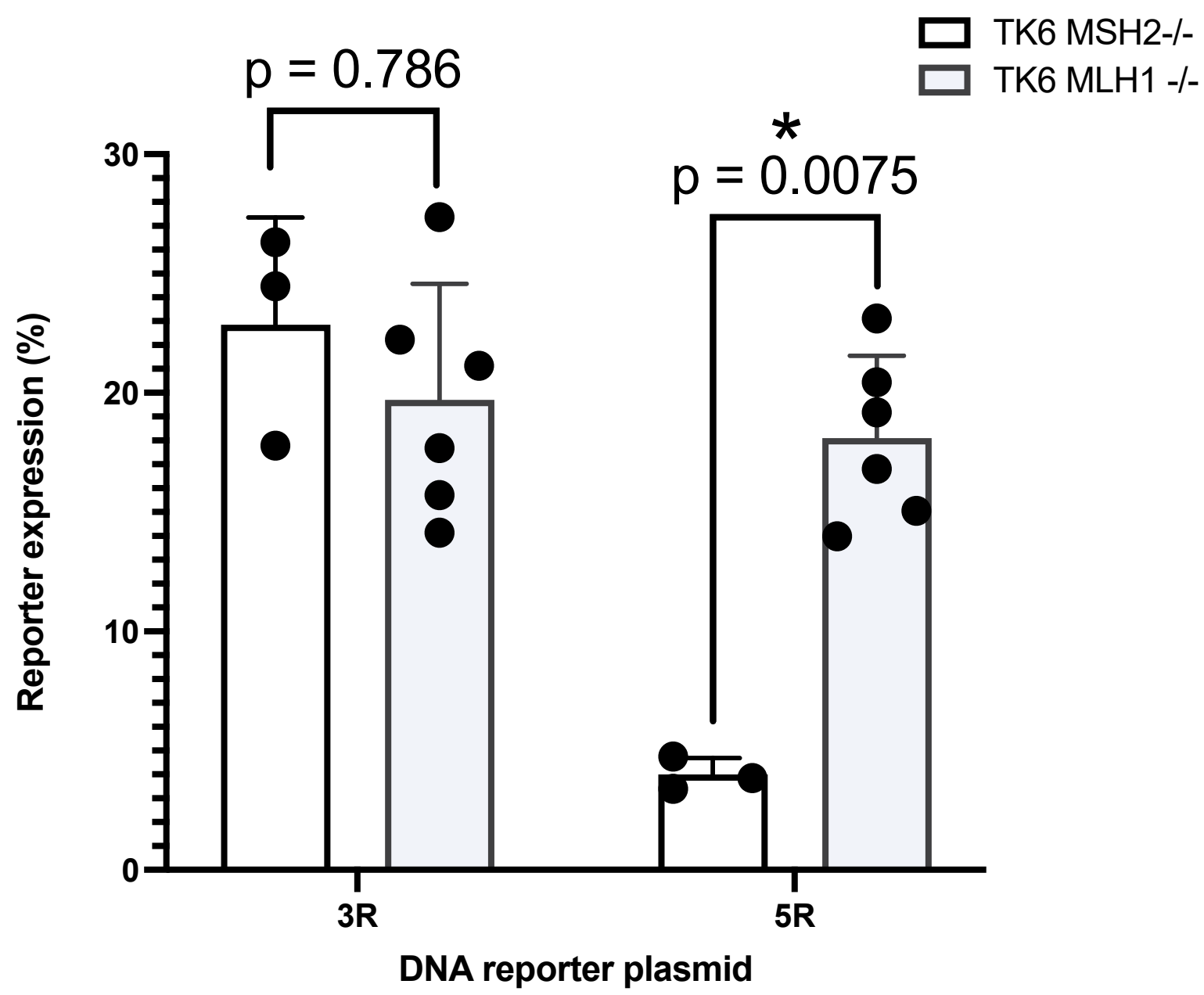

Figure S.4: MMR capacity of MutS and MutL component knockouts in TK6 cells with an incorporated ribonucleotide. An ordinary one-way ANOVA run with Tukey's multiple comparisons test revealed statistical significance of reporter expression of mOrangeGG5R in TK6 MSH2$^{-/-}$ cells compared to TK6 MLH1$^{-/-}$ cell. Each dot represents reporter expression (%) means, and the error bars represent standard deviation. * $p<0.05$

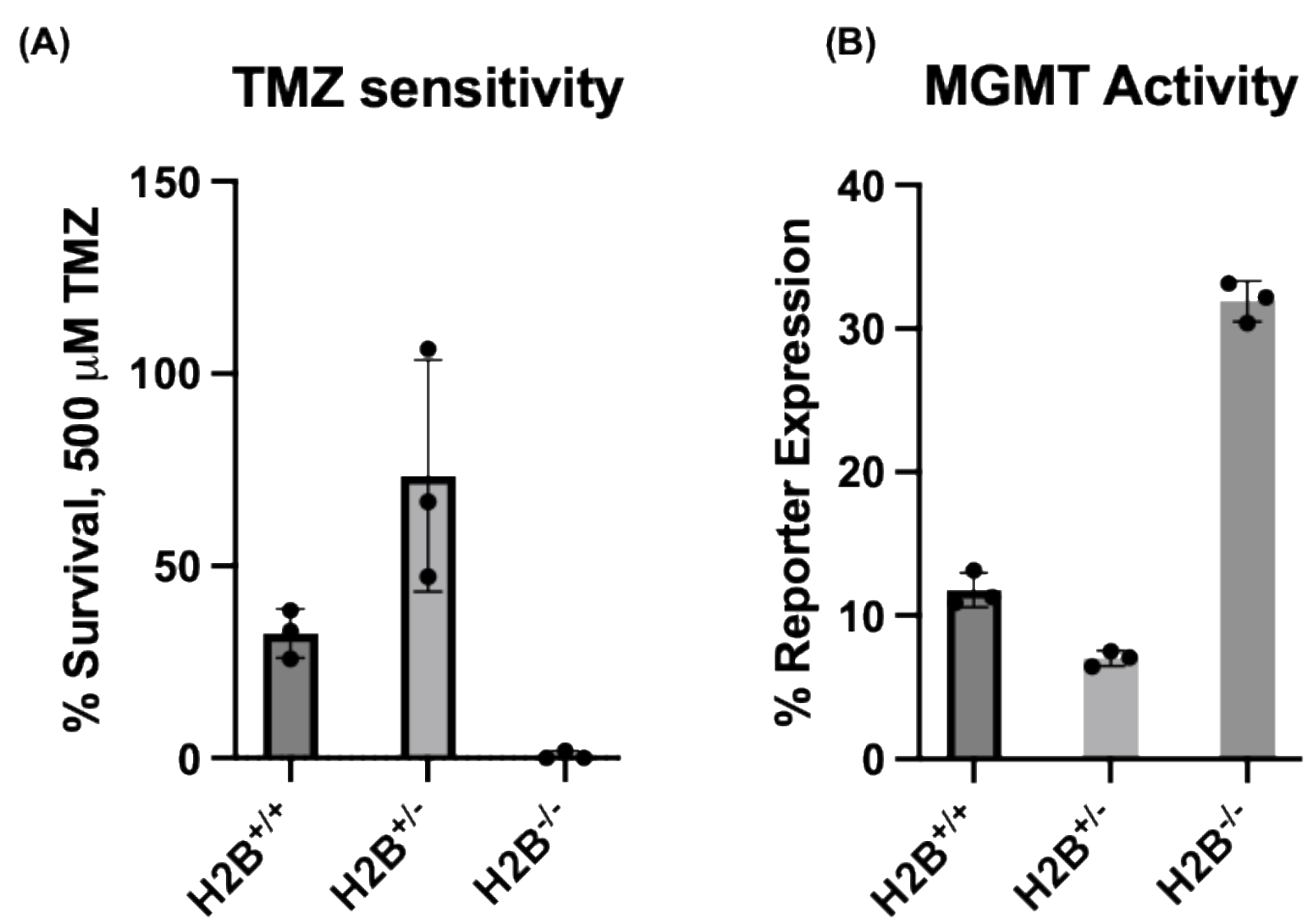

Figure S.5: TMZ sensitivity and MGMT activity measured in RNaseH2 KO MEFs

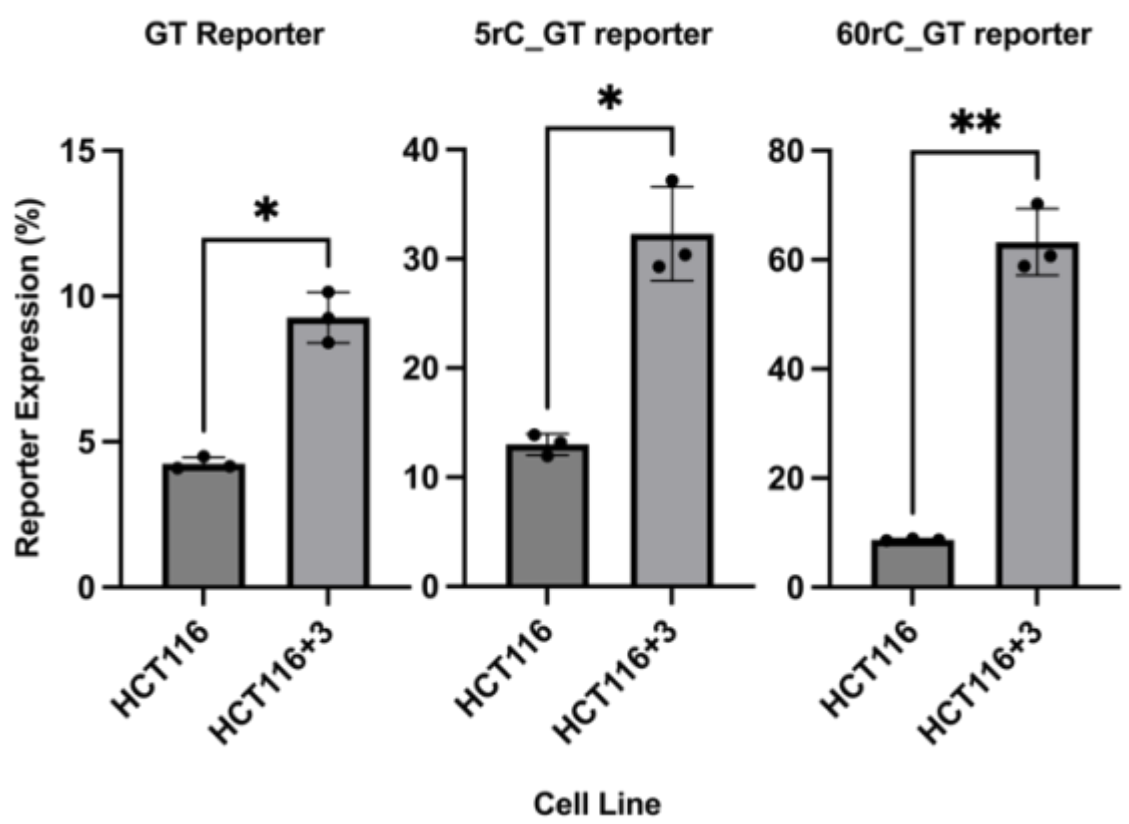

Figure S.6: A G:T mismatch with a 5' ribonucleotide 5 nucleotides away from the mismatch (5rC_GT) is repaired with slightly greater efficiency in MMR deficient HCT116 cells that the reporter plasmid with a ribonucleotide embedded 65 nucleotides away in the in 5' direction (60rC_GT). Reporter expression is compared between cell lines for each reporter by paired t-test. Each data point represents an independent biological replicate, and error bars show the standard deviation. Statistical significance is reported as ns (not significant), * p<0.05; ** p<0.005

Table S1: Reporter expression of mOrange_GG, mOrange_GG3R, and mOrange_GG5R in TK6 cells

Reporter	TK6 WT								
	TF1	TF2	TF3	TF4	TF5	TF6	TF7	TF8	TF9
GG	1.74	2.12	2.10	1.85	1.71	2.09	3.10	6.34	5.89
3R	72.51	74.16	74.94	85.10	69.10	74.94	85.83	94.06	80.71
5R	70.32	72.46	64.80	81.09	77.23	64.75	80.77	80.59	83.04

Reporter	TK6 RNaseHIIA$^{-/-}$			TK6 MSH2$^{-/-}$		
	TF1	TF2	TF3	TF1	TF2	TF3
GG	2.34	1.69	1.64	0.85	1.20	0.87
3R	74.70	58.43	65.56	17.78	26.32	24.47

5R	55.98	43.93	51.57	3.40	4.75	3.85

Reporter	TK6 MLH1$^{-/-}$					
	TF1	TF2	TF3	TF4	TF5	TF6
GG	0.70	0.41	0.80	0.81	1.11	1.06
3R	21.14	14.14	17.68	15.71	22.23	27.36
5R	23.11	15.06	20.45	13.99	16.81	19.18

Table S2: Reporter expression of mOrange_GG, mOrange_GG3R, and mOrange_GG5R in U2OS cells

Reporter	**U2OS NT**		
GG	35.326	40.190	46.435
3R	48.938	47.225	56.672
5R	56.842	70.610	63.675

Reporter	**U2OS MLH1(4)$^{-/-}$**			**U2OS MLH1(7)$^{-/-}$**		
GG	2.420	2.756	2.656	2.101	2.287	2.289
3R	4.545	5.879	5.635	8.352	7.347	8.490
5R	2.568	2.940	2.690	2.104	2.697	3.131

Reporter	**U2OS MSH6(7)$^{-/-}$**			**U2OS MSH6(11)$^{-/-}$**		
GG	2.778	3.577	2.347	3.184	2.873	2.970
3R	8.395	8.608	10.621	7.806	7.991	8.008
5R	2.697	2.505	3.111	2.705	2.850	2.970

Table S3: Reporter expression of mOrange_GG, mOrange_GG3R, and mOrange_GG5R in cancer cell lines

Reporter	**HCT116**				**HCT15**		
GG	3.103	1.593	2.592	1.403	0.915	0.708	0.745
3R	15.692	10.425	11.969	9.611	8.012	5.509	6.594
5R	9.726	4.244	4.981	4.619	2.997	1.210	2.017

	**SKOV3**				**DU145**		
GG	1.171	0.492	1.594	0.569	0.932	2.496	1.318
3R	5.870	4.007	4.816	4.592	18.664	22.290	16.587
5R	1.002	0.750	1.164	0.550	6.378	7.866	4.994

	U251				U2OS	
GG	53.81	48.77	30.87	36.78	44.89	28.22
3R	111.41	88.00	62.78	82.86	67.02	55.98
5R	130.17	109.10	59.30	95.95	72.31	72.04
